# Targeting PGK1 as a Novel strategy to regulate the sensitivity of HER2 positive gastric cancer to lapatinib

**DOI:** 10.3389/fphar.2025.1530492

**Published:** 2025-07-25

**Authors:** Xiaochen Ni, Kaiyuan Zhang, Xueru Huang, Mingsi Zhang, Jianing Guo, Wei Fan, Chuhang Wang, Zhongyan Du, Tao Jiang, Guangji Zhang

**Affiliations:** ^1^School of Basic Medical Sciences, Zhejiang Chinese Medical University, Hangzhou, Zhejiang, China; ^2^School of Sport, Loughborough University, Loughborough, Leicestershire, United Kingdom; ^3^Zhejiang Key Laboratory of Blood-Stasis-Toxin Syndrome, Zhejiang Chinese Medical University, Zhejiang, China; ^4^ Traditional Chinese Medicine “Preventing Disease”Wisdom Health Project Research Center of Zhejiang, Zhejiang, China

**Keywords:** glycolysis, PGK1, lapatinib, HER2 gastric cancer, lactate

## Abstract

HER2 is amplified in approximately 20% of gastric cancers, and these patients exhibit a favorable response to trastuzumab treatment. Lapatinib, as a HER2-targeted drug, demonstrates potent inhibitory effects on HER2-addicted N87 gastric cancer cells. However, lapatinib has not shown significant advantages in clinical trials. Our study revealed that the expression of the key glycolysis gene PGK1 negatively correlates with the sensitivity of tumor cells to lapatinib. Both genetic regulation of PGK1 and pharmacological inhibition of lactate secretion can enhance the inhibitory effect of lapatinib on N87 cells, whereas overexpression of PGK1 attenuates the efficacy of lapatinib. Modulating PGK1 expression in N87 cells exposed to lapatinib affects the activation level of AKT, a downstream effector of HER2, and consequently influences the viability of N87 cells. This study indicates that regulating the expression levels of PGK1 impacts the sensitivity of HER2-positive gastric cancer to lapatinib, and potentially serving as a therapeutic strategy for HER2-positive gastric cancer patients who do not respond to lapatinib.

## 1 Introduction

Gastric cancer is one of the most common cancers worldwide and the second leading cause of cancer-related deaths. Approximately one million new cases of cancer are diagnosed annually, and about 800,000 patients die from gastric cancer (Bray et al., 2024). Due to the nonspecific or even asymptomatic nature of early gastric cancer, most patients are diagnosed at an advanced stage (Smyth et al., 2020), where they have typically lost the opportunity for surgery, and can only rely on systemic drug treatment (Lordick et al., 2022). Among patients diagnosed with gastric cancer at an early stage, the 5-year overall survival rate is approximately 60%, while it is less than 5% for stage IV patients (Sastre et al., 2006). Among patients with metastatic gastric cancer, the median survival time for those receiving chemotherapy and other therapies is 5–9 months. Therefore, it is imperative to promote the development of treatment strategies for advanced gastric cancer, such strategies could prevent hundreds of thousands of patient deaths annually.

Erb-b2 receptor tyrosine kinase 2 (ERBB2/HER2) regulates cell survival and proliferation by activating the PI3K/AKT/mTOR signaling pathway, contributing to adverse pathological features. HER2 is overexpressed in approximately 20% of gastric cancer patients and may be associated with poor prognosis (Mezynski et al., 2021). In stage III gastric cancer patients with HER2 amplification, combination therapy with HER2 monoclonal antibodies can increase overall survival by 2.7 months (Bang et al., 2010). Therefore, trastuzumab, an anti-HER2 agent, combined with chemotherapy is the first-line treatment for patients with advanced gastric cancer who have high HER2 expression. However, the clinical efficacy of this treatment regimen is only 47%. Furthermore, acquired resistance often develops within 1 year in patients who initially respond to trastuzumab (Bang et al., 2010). Consequently, there is a clinical need for more anti-HER2 therapies to address the frequent occurrence of drug resistance in cancer patients, as these patients may soon find themselves with no effective treatment options. However, a study revealed that lapatinib, another HER2-targeted drug, failed to prolong survival in the intended patient population (Satoh et al., 2014). Therefore, investigating the mechanisms of drug sensitivity and resistance may lead to new treatment strategies to improve the sensitivity of HER2-positive patients to lapatinib.

Enhanced glycolysis, a hallmark metabolic feature of tumors, directly contributes to the avid glucose consumption and increased lactate production by tumor cells, which is associated with various malignant phenotypes of tumors. The role of lactate in promoting tumor progression and sustaining malignant phenotypes has been documented since the 1920s. Notably, lactylation, a recently identified epigenetic modification, has expanded our understanding of lactate’s functions in tumors beyond merely serving as an energy source. It has been demonstrated that lactylation of histone H3 lysine 18 (H3K18la) is crucial for tumorigenesis (Yu et al., 2021), progression, immune evasion (Xiong et al., 2022), and metabolic reprogramming (Yang et al., 2023). A recent pivotal study showed that lactylation of NBS1 is essential for gastric cancer resistance to radiotherapy and chemotherapy (Chen et al., 2024). This study demonstrates that the level of PGK1 protein is directly correlated with the sensitivity of HER2-dependent gastric cancer cells to lapatinib, providing a potential strategy for the application of lapatinib in patients with gastric cancer who have high HER2 expression.

## 2 Methods and materials

### 2.1 TCGA data collection and analysis

Clinical information and RNA sequencing data were obtained from the STAD database of TCGA, with inclusion criteria as follows: (a) samples diagnosed with GC; (b) samples with complete clinical information, including at least survival time, survival status, age, and gender; (c) for paired samples, only one sample per pair was retained. Exclusion criteria were as follows: (a) normal tissue samples; (b) samples without complete clinical information. A total of 333 STAD samples obtained from the database were defined as the training cohort. The hypoxia-glycolysis-lactate-related gene (GLRG) set was obtained from a previously published study (Zhang et al., 2023), as detailed in Supplementary Material S1. Firstly, univariate Cox regression analysis was conducted on TCGA data to identify prognostic-related genes in GC. By intersecting the GC prognostic-related genes with GLRGs, 141 prognostic-related GLRGs were obtained. Subsequently, the R package “ConsensusClusterPlus” was used to perform consensus clustering based on the expression matrix of the prognostic-related GLRGs.

### 2.2 Establishment and validation of a prognostic risk model based on GLRGs

We conducted Least Absolute Shrinkage and Selection Operator (LASSO) analysis to construct a risk feature model for assessing the prognostic predictive value of GLRGs in GC. To optimize the penalty parameter (λ) and prevent overfitting, we implemented 10-fold cross-validation, selecting the λ value that minimized the cross-validated partial likelihood deviance. The final model retained 36 genes with non-zero coefficients. Patients were divided into high-risk and low-risk groups based on the median risk score. Kaplan-Meier survival curves were used to predict the prognosis of the two patient groups. Finally, we validated the prognostic risk model integrating the GSE14210, GSE15459, GSE22377, GSE29272, GSE51105 and GSE62254 dataset via the Kaplan-Meier Plotter website1. To evaluate whether the risk model constructed using 36 GLRGs could potentially serve as an independent prognostic factor for GC, we explored the correlation between risk scores and clinical features, and assessed the independence of the constructed risk model through subgroup analysis and regression analysis.

### 2.3 Analysis of hub genes

Hub genes were identified by the cytoHubba plugin in Cytoscape. Five algorithms, namely, Degree, EPC, MCC, Radiality, and Closeness, were employed to obtain 10 hub genes each. The genes obtained from these five algorithms were then intersected to determine the final hub genes. The association scores between the hub genes and glycolysis were retrieved in GeneCards. By combining the scores from the algorithms and the association scores, the hub gene with the highest score was selected as PGK1. Pan-cancer mRNA expression analysis of PGK1 was conducted using data from TCGA and GTEx. In the TCGA-STAD database, the top 30 genes positively correlated with PGK1 expression were identified, and a clustered heatmap was generated for visualization by the Xiantao tool. Finally, Gene Ontology (GO) and Kyoto Encyclopedia of Genes and Genomes (KEGG) enrichment analyses were performed on these 30 PGK1-positively correlated genes.

### 2.4 Drugs response analysis

GSCALite (http://bioinfo.life.hust.edu.cn/web/GSCALite/) (Liu et al., 2018) is used for analyzing TCGA datasets and drug resistance data from GDSC (https://www.cancerrxgene.org/) and CRTP (http://portals.broadinstitute.org/ctrp/). We used GSCALite to analyze the pan-cancer drug sensitivity of PGK1 in the GDSC and CRTP databases. Based on the retrieved datasets, chemotherapy drugs approved by the Food and Drug Administration (FDA) that are associated with PGK1 expression were identified in DrugBank (https://go.drugbank.com/drugs). Finally, the results were visualized using Cytoscape (http://www.cytoscape.org) (Smoot et al., 2011).

### 2.5 Cell culture

The NCI-N87 (Catalog No.: CL-0169), HGC-27 (Catalog No.: CL-0107), AGS (Catalog No.: CL-0022), and MKN-45 (Catalog No.: CL-0292) cell lines were obtained from Wuhan Pricella Biotechnology Co., Ltd. According to the company’s cultivation guidelines, N87, HGC-27, and MKN-45 cells were cultured in RPMI-1640 medium (10% FBS). AGS cells were cultured in Ham’s F-12 medium (10% FBS). When the cell confluence reached 90%, the cells were passaged at a ratio of 1:3, and the medium was replaced every 2–3 days. The incubator was set at 37°C, 5% CO_2_, and saturated humidity.

### 2.6 Reagents and antibodies

The culture medium (Catalog No.:11875119) and fetal bovine serum (Catalog No.:A5669701) were obtained from Gibco. CCK-8 (Catalog No.:C0039), DMSO (Catalog No.:ST038), crystal violet (Catalog No.:C0121), and EDU kit (Catalog No.:C0075) were obtained from Beyotime Biotechnology. L-Lactic acid (Catalog No.:L1750) and lapatinib (Catalog No.:CDS022971) were obtained from Sigma-Aldrich. BCECF AM (Catalog No.:B1170) was acquired from Thermo Fisher Scientific. The L-Lactic acid kit (Catalog No.:E-BC-K044-M) was obtained from Elabscience Biotechnology Co., Ltd. P-HER2 (Catalog No.: 2243), HER2 (Catalog No.:2165), P-AKT (Catalog No.:4060), AKT (Catalog No.:9272), and pgk1 (Catalog No.:68540) were sourced from Cell Signaling Technology, Inc. GAPDH was obtained from Proteintech Group, Inc.

### 2.7 Cell proliferation and viability assays

Cell proliferation or toxicity tests were conducted by the WST-8-based cell counting kit-8 (CCK-8) assay. Approximately 10,000 cells/well were added to a 96-well plate and incubated overnight before a specified concentration of lapatinib was added. After exposing gastric cancer cells to the specified concentration of the drug for 24 h, the drug-containing medium was removed, and serum-free medium containing 10% CCK-8 solution was added. The plate was then incubated at 37°C for an additional hour. Subsequently, the absorbance was measured at 450 nm using a microplate reader.

For long-term cell viability assays, culture plates corresponding to the number of days required were prepared, and 200 μL of medium was added to each well to support cell proliferation for up to 5 days. The same tube of cell suspension was used to seed the 96-well plate, maintaining a cell count of 1,500–3,000 cells/well, and a specified concentration of lapatinib was added. The wells were labeled with numbers one to five, and absorbance values were measured using the CCK-8 assay on the corresponding days. These absorbance values were then converted into cell counts using a standard curve.

### 2.8 Western blot

Following seeding in a cell culture dish, gastric cancer cells were treated with lapatinib at designated concentrations upon reaching 70%–80% confluence. After two washes with phosphate-buffered saline (PBS), cells were lysed using RIPA buffer supplemented with phosphatase inhibitors. The lysates were centrifuged at 15,000 × g for 30 min at 4°C, and the supernatant was collected. The supernatant was divided into two aliquots: one was mixed with loading buffer and boiled at 95°C for 5 min, while the other was subjected to protein quantification via the bicinchoninic acid (BCA) assay. Proteins (20 μg per lane) were separated by SDS-PAGE and transferred onto a polyvinylidene difluoride (PVDF) membrane using a wet transfer system. Membranes were blocked with 5% skimmed milk for 1 h at room temperature, followed by incubation with primary antibodies overnight (approximately 14 h) at 4°C. HRP-conjugated secondary antibodies were incubated with the membranes for 1 h at room temperature. Image acquisition was performed by the Bio-Rad imaging system, and the protein bands were normalized and analyzed based on grayscale values.

### 2.9 Transfection

The lentiviruses for overexpression and small interfering RNAs (siRNAs) were designed and packaged by GENECHEM (Shanghai, China), and the infection protocol was performed according to the manufacturer’s instructions. Gastric cancer cells were seeded into 6-well plates at a density corresponding to 15%–20% confluence. A mixture of medium, virus, and infection reagent (provided by GENECHEM) was prepared at the recommended multiplicity of infection (MOI) and added to each well (1 mL/well), followed by incubation for 16 h. The medium containing the virus was then removed and replaced with fresh complete medium. After 48 h of culture, when cells exhibited fluorescence, puromycin (2 μg/mL) was added to the medium to select stably transfected cells and maintained for 48 h. When fluorescence was observed in all cells, the polyclonal cell line was established.

### 2.10 Colony formation

Cells were seeded in 6-well plates at 800 cells/well and incubated overnight to adhere, followed by treatment with lapatinib at specified concentrations. After 24 h of drug exposure, the medium was replaced with fresh medium to remove residual drug. Cells were cultured for 14 days, with medium refreshed every 3 days. When colonies in the control group reached >60 cells (defined as a cluster), cells were fixed with 4% paraformaldehyde for 20 min and stained with 0.1% crystal violet for 30 min.

### 2.11 EdU assay

EdU, a thymidine analog, can incorporate into DNA during cell proliferation and is therefore employed for precise detection of cellular proliferation status. Gastric cancer cells were cultured in a 6-well plate until they reached approximately 60% confluence. The cells were washed twice with PBS and then fixed with 4% paraformaldehyde for 15 min. Following this, the cells were washed three times with PBS containing 3% BSA, each time for 5 min. Subsequently, the cells were permeabilized with PBS containing 0.3% Triton X-100 for 15 min and washed again three times, each time for 5 min. The click reaction mixture was prepared strictly according to the manufacturer’s instructions and used to fully cover the cells. The cells were then incubated in the dark at room temperature for 30 min. Afterwards, the cells were washed three times, each time for 5 min. The staining of cell nuclei was accomplished using DAPI. Image acquisition was performed using the KEYENCE BZ-X800E imaging system, and data analysis was conducted with ImageJ.

### 2.12 Lactate detection

The determination of lactic acid content was accomplished through the commercialized reagent kit. In brief, lactic acid dehydrogenase (LDH) catalyzed the dehydrogenation of lactic acid to produce pyruvate, thereby converting NAD^+^ into reduced nicotinamide adenine dinucleotide (NADH). Here, N-methylphenazine methosulfate (PMS) served as a hydrogen donor, enabling the reduction of nitroblue tetrazolium chloride (NBT) into a purple color product. The absorbance of this colored product at 530 nm exhibited a linear relationship with the lactic acid content. Cells were cultured in 96-well plates, each containing 100 μL of medium. The operational procedure involved aspirating 5 μL of the medium as a sample, adding 120 μL of reaction working solution, incubating at 37°C for 10 min, subsequently adding 180 μL of reaction stop solution, and finally measuring the absorbance at 530 nm by a microplate reader. It is crucial to note that fetal bovine serum contains lactic acid, necessitating the establishment of a negative control to eliminate potential interference.

### 2.13 Lactate influx experiment

The lactate influx experiment was conducted with reference to a previously established protocol (Wang et al., 2023). The BCECF AM probe was utilized for real-time monitoring of intracellular pH levels. The BCECF AM probe can be excited at 495 nm and 440 nm, with emission detected at 535 nm. The pH value was characterized by the fluorescence ratio obtained from excitation at 495 nm and 440 nm. Cells were pre-cultured in 96-well plates and subjected to drug treatment after they had recovered their morphology. The BCECF AM probe was diluted to a concentration of 1 μM using Hanks' Balanced Salt Solution (HBSS) and added to the 96-well plates. The cells were then further incubated in the dark for 30 min, followed by two washes with serum-free medium. Cellular fluorescence was detected using a multi-functional microplate reader, with measurements taken every 5 s in dual-laser mode. Lactate at a concentration of 13 mM was added at 17.6 s through the multi-channel dispenser integrated within the microplate reader (initial medium volume of 50 μL, with an additional 50 μL of lactate-containing medium added, resulting in a final lactate concentration of 6.25 mM).

### 2.14 Establishment of xenograft tumor models

Tumor cells in the logarithmic growth phase were digested and resuspended in PBS, and the cell density was adjusted to 1 × 10^7^ cells/ml through counting. The tumor cells were then injected subcutaneously into the right flank of nude mice, with an injection volume of 2 × 10^6^ cells in 200 μL per mouse. Approximately 2 days later, noticeable subcutaneous growths were observed in the mice, at which point the mice were started on oral gavage with either lapatinib (80 mg/kg) or normal saline. The tumor size and mouse body weight were recorded every 2 days. The experiment was immediately terminated when the volume of the subcutaneous tumor in any mouse reached 2000 mm^3^ or the length in any dimension reached 20 mm (as occurred with Mouse #2 in the control group). These mice were housed in a barrier facility throughout the experiment and received appropriate welfare. All procedures were conducted in accordance with the regulations of the Animal Experiment Ethics Committee of Zhejiang Chinese Medical University.

## 3 Results

### 3.1 TCGA and GEO data reveal high expression of GLRGs as a poor prognostic factor for GC

**FIGURE 1:**
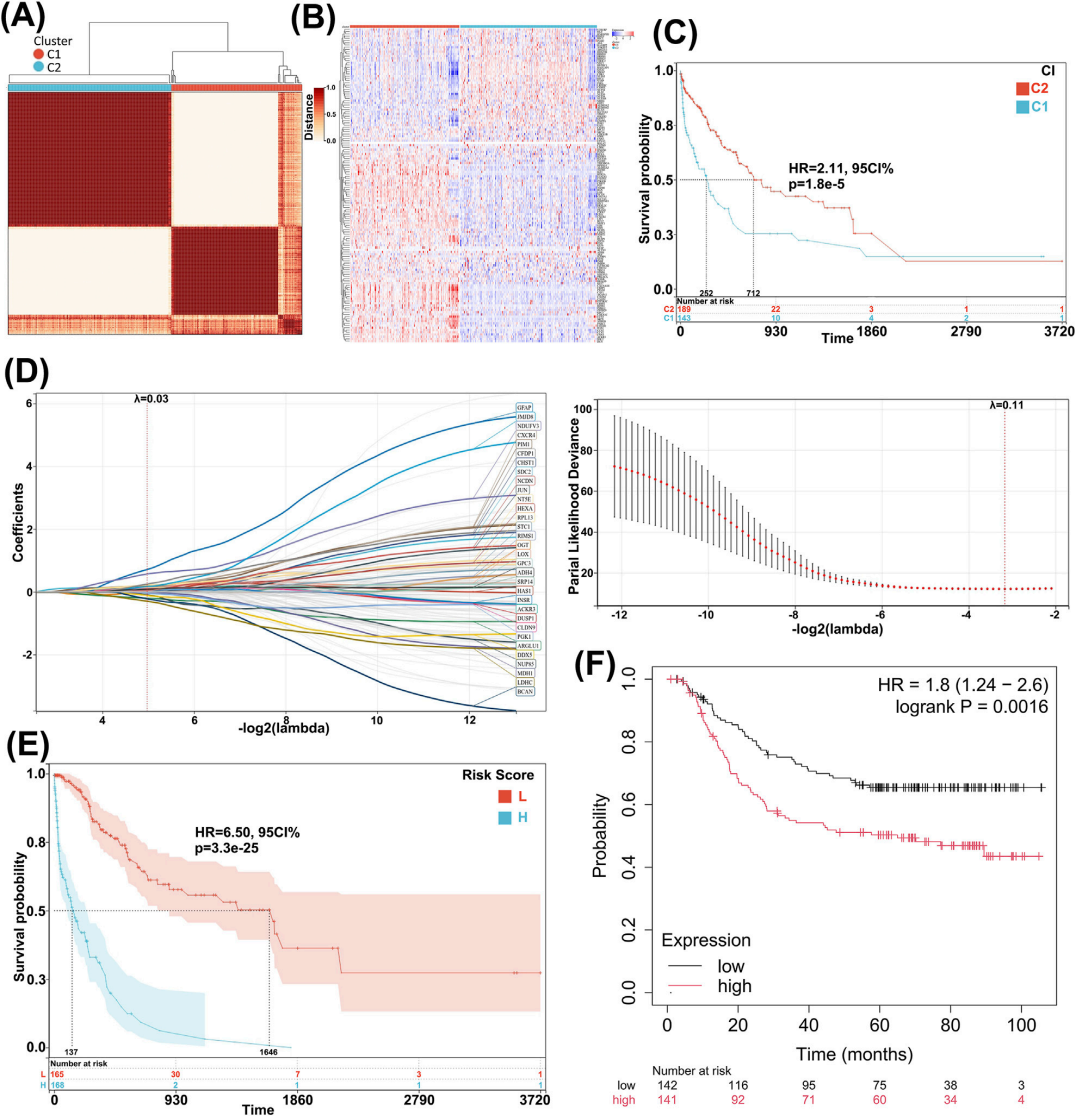
Identification and validation of prognosis-related GLRGs. **(A)** Consensus clustering yields the optimal value when K = 2, dividing patients into two subgroups, C1 and C2. **(B)** The heatmap displays the mRNA expression of GLRGs in the two subgroups, with colors ranging from blue to red representing increasing gene expression levels. **(C)** Survival curves of GC patients in the two subgroups demonstrate differences in prognosis. **(D)** The risk model is constructed in the training cohort, and 36 independent prognostic signature genes are obtained through LASSO analysis at the minimum λ value. **(E)** Survival curves of GC patients are constructed based on the high- and low-risk groups defined by the 36 genes. **(F)** KM curves of the 36 genes in the GSE62254 validation cohort are presented.

Cox analysis of gastric adenocarcinoma data from the TCGA database yielded 5,986 prognosis-related genes. Subsequently, 714 GLRGs were collected through literature review, and the intersection of these two sets resulted in 141 GC prognosis-related GLRGs. Based on these 141 prognostic genes, consensus clustering was employed, with K = 2 yielding the best clustering stability (Figure 1A). One hundred eighty-nine patients were classified into the first group, and one hundred forty-three patients were classified into the second group. The heatmap (Figure 1B) displayed the expression levels of GLRGs in the two groups, revealing significant expression differences between C1 and C2. Furthermore, KM survival curve analysis showed that the overall survival of patients in the second group was significantly higher than that of patients in the first group (P < 0.001; Figure 1C). These results indicate a poorer prognosis for the high-expression GLRGs group. Next, we constructed a risk signature model to assess the prognostic predictive value of GLRGs in GC. Potential genes for establishing the risk model were screened through LASSO analysis, ultimately identifying 36 genes with the optimal λ value (λ = 0.0318) (Figure 1D). The risk model, constructed based on these 36 GLRGs identified by LASSO analysis, successfully stratified GC patients into high-risk and low-risk groups. The overall survival rate was higher in the low-risk group than in the high-risk group (Figure 1E). Subsequently, the GSE62254 dataset was used as a validation cohort for KM survival curve analysis, and the validation results showed that in this dataset, the survival duration of the high-expression gene group was significantly shorter than that of the low-expression group (Figure 1F).

### 3.2 Detection of independence in risk model construction

**FIGURE 2:**
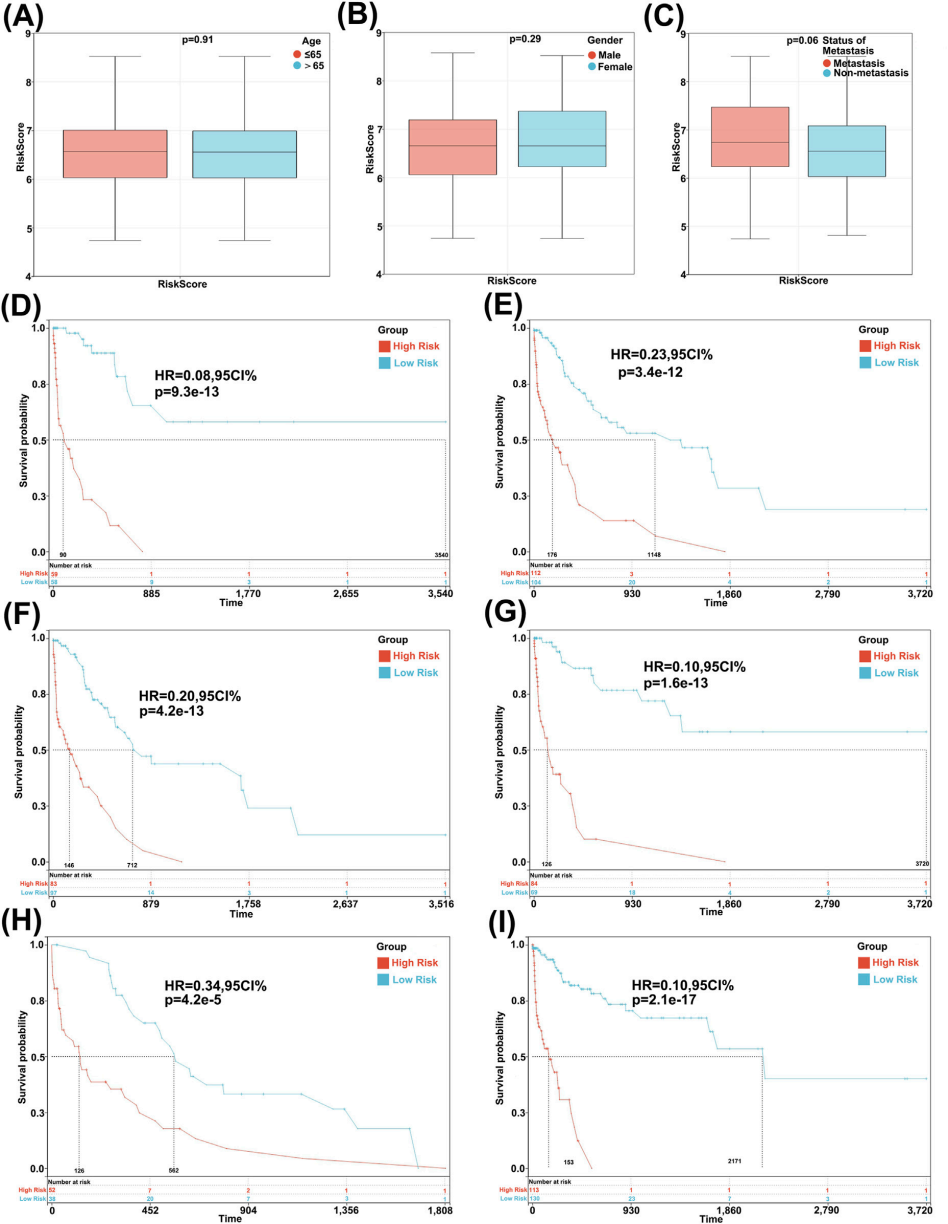
Analysis of characteristic and independence of the 36-Gene risk prognostic model. **(A)** Association between risk score and gender characteristics. **(B)** Association between risk score and age characteristics. **(C)** Association between risk score and tumor metastasis characteristics. Survival curves of the patient risk prognostic model after regrouping based on age **(D,E)**, gender **(F,G)**, and metastasis **(H,I)**.

We examined the relationship between the risk score and clinical characteristics and evaluated the independence of the constructed risk model through subgroup analysis and regression analysis. Patients of different ages (Figure 2A), genders (Figure 2B), and metastatic statuses (Figure 2C) did not differ significantly in risk scores, indicating no correlation between the risk score and clinical characteristics. Furthermore, when patients were regrouped based on age (Figures 2D,E), gender (Figures 2F,G), and metastatic status (Figures 2H,I), the risk model consistently demonstrated robust predictive performance, with patients having lower risk scores demonstrating better prognosis. These results suggest that the risk model constructed using 36 GLRGs genes exhibits excellent independence in predicting the prognosis of gastric cancer (GC).

### 3.3 Identification of hub genes and exploration of Co-expressed genes

**FIGURE 3:**
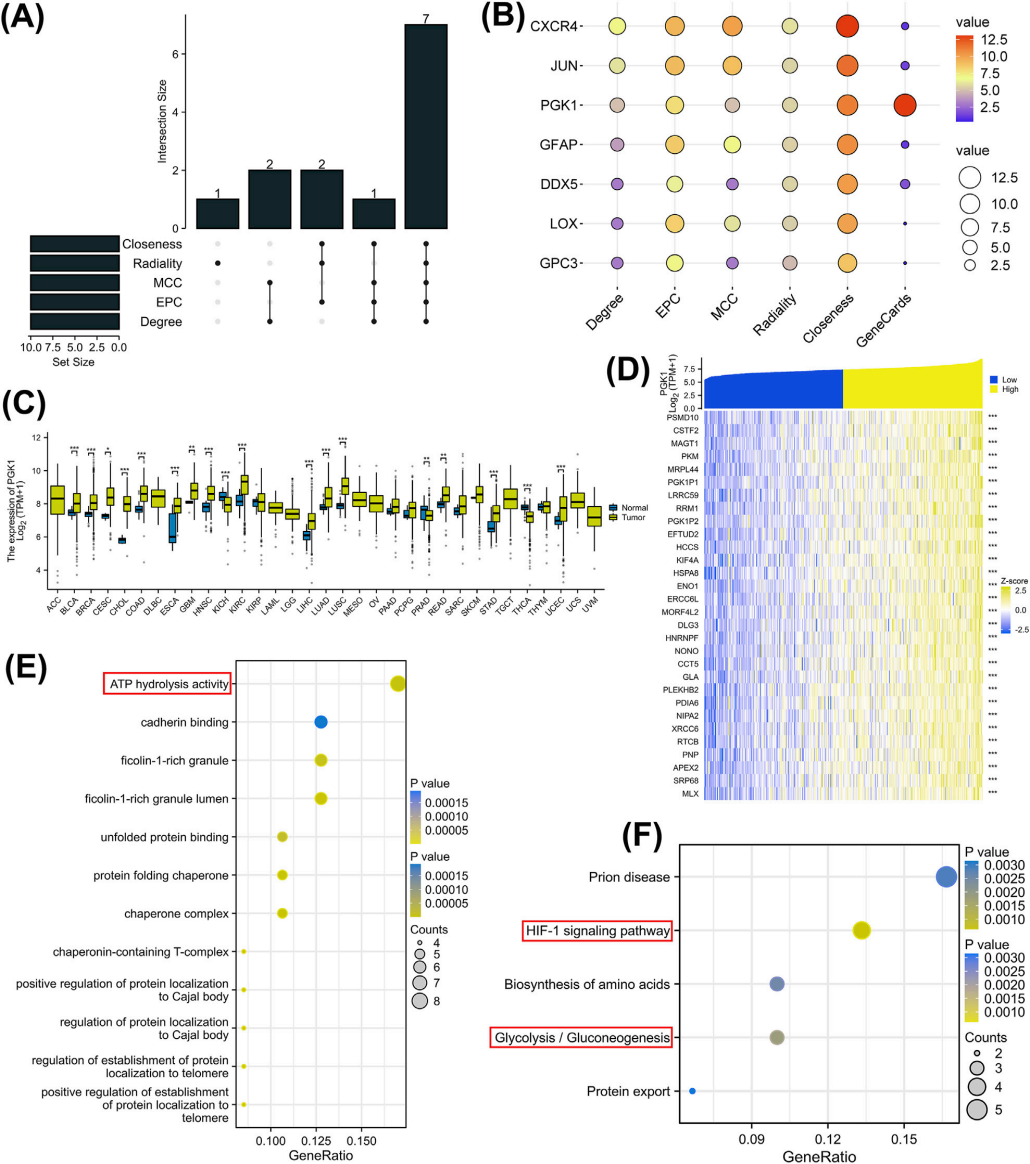
Further analysis of prognosis-related GLRGs. **(A)** Upset plot showing the intersection of 10 hub genes obtained using five algorithms. **(B)** Heatmap of scores for 7 hub genes across five algorithms. **(C)** Expression of PGK1 mRNA between tumor and normal tissues from TCGA and GTEx (N = 15,776). **(D)** Heatmap of expression for the top 30 co-expressed genes positively correlated with PGK1 expression in STAD. **(E,F)** GO and KEGG enrichment analyses of the 30 co-expressed genes of PGK1.

Five cytohubba algorithms were applied to analyze the 36 genes, and an upset plot was utilized to identify seven hub genes (Figure 3A). Subsequently, a score plot was employed to calculate their comprehensive scores. The associations between these seven hub genes and glycolysis-lactate fermentation were searched in Genecards, yielding their relevance scores, with PGK1 emerging as the highest-scoring gene (Figure 3B; Table 1). Further analysis of PGK1, the top-ranked gene, revealed significant upregulation in BLCA, BRCA, CESC, CHOL, COAD, ESCA, GBM, HNSC, KICH, KIRC, LGG, LIHC, LUAD, LUSC, PRAD, STAD, THCA, and UCEC based on TCGA and GTEx datasets (P < 0.05) (Figure 3C). We then identified co-expressed genes related to PGK1 in TCGA. A heatmap displayed the top 30 co-expressed genes positively correlated with PGK1 in STAD (Figure 3D). Subsequent GO and KEGG enrichment analyses were conducted on these co-expressed genes. GO analysis implicated biological pathways such as ATP hydrolase activity and cadherin binding (Figure 3E). KEGG results indicated that PGK1 co-expressed genes are involved in GC through the regulation of glycolysis/gluconeogenesis, HIF-1α signaling, and ATP hydrolase activity pathways (Figure 3F).

**TABLE 1:** The 7 hub genes rank in cytoHubba and GeneCards.

Gene	Degree	EPC	MCC	Radiality	Closenesee	GeneCards
CXCR4	7	9.34	10	5.89	13.08	0.999
JUN	6	9.04	9	5.50	11.62	1.37
PGK1	5	7.862	5	5.46	11.12	13.01
GFAP	4	8.528	7	5.41	10.62	1.10
DDX5	3	6.326	3	5.46	10.17	1.81
LOX	3	8.385	6	5.33	9.95	0.35
GPC3	3	6.992	3	4.80	8.85	0.36

### 3.4 Pan-cancer sensitivity analysis of PGK1-Related drugs

**FIGURE 4:**
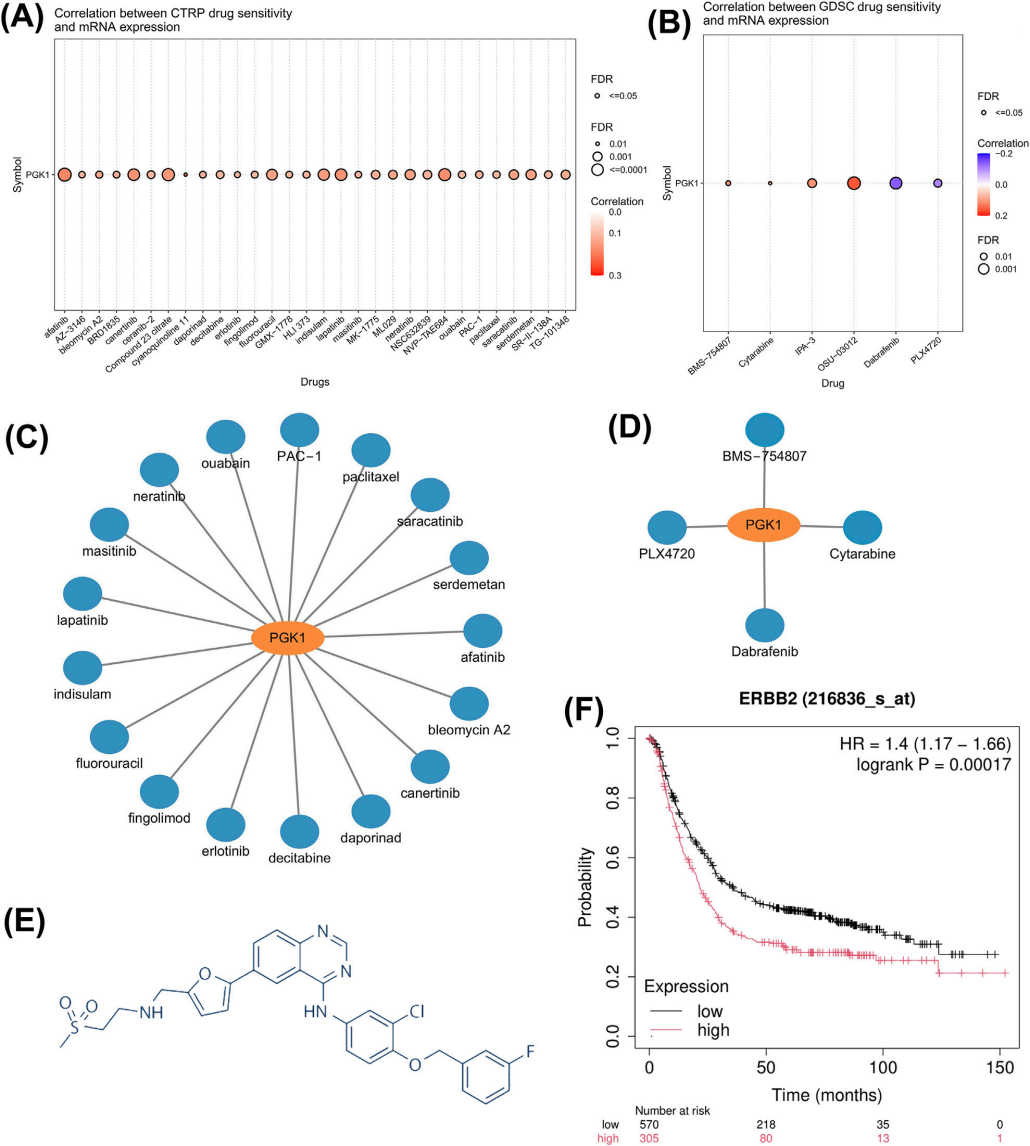
Drug sensitivity of PGK1-related drugs in pan-cancer tissues. **(A)** Relationship between CTRP drug sensitivity and PGK1 mRNA expression. **(B)** Correlation between GDSC drug sensitivity and PGK1 mRNA expression. **(C)** FDA-approved PGK1-related CTRP chemotherapy sensitivity drugs. **(D)** FDA-approved PGK1-related anticancer drugs from GDSC drug sensitivity. **(E)** The molecular formula of lapatinib. **(F)** Combined prognostic survival curves for the HER2 low-expression group versus the high-expression group in gastric cancer GEO datasets: GSE14210, GSE15459, GSE22377, GSE29272, GSE51105, and GSE62254.

The CTPR dataset indicates a correlation between PGK1 mRNA expression levels and drug sensitivity. The top three drugs positively correlated with PGK1 expression are afatinib, lapatinib, and canertinib (Figure 4A, P < 0.0001). According to GDSC drug sensitivity data, the top three drugs showing a positive correlation with PGK1 expression are OSU-03012, IPA-3, and BMS-754807, while drugs negatively correlated with PGK1 expression include Dabrafenib, PLX4720, and Cytarabine (Figure 4B, P < 0.0001). Based on DrugBank data, 17 PGK1-related antitumor drugs approved by the FDA in CTPR (Figure 4C) and 4 in GDSC (Figure 4D) were plotted. Lapatinib is a HER2 inhibitor, initially approved for the treatment of advanced or metastatic breast cancer with HER2-positive pathology (Figure E). Amplification of HER2 directly contributes to poor prognosis in gastric cancer patients (Figure 4F), prompting the use of lapatinib in patients with advanced gastric cancer that is HER2-positive. However, a preliminary clinical study indicated no advantage in treating HER2-amplified advanced or metastatic gastric cancer patients with chemotherapy combined with lapatinib (LBA4001, LOGiC study). Consequently, we decided to investigate the correlation between PGK1 and the sensitivity of HER2-positive gastric cancer to lapatinib.

### 3.5 NCI-N87 is HER2-Addicted and sensitive to lapatinib

**FIGURE 5:**
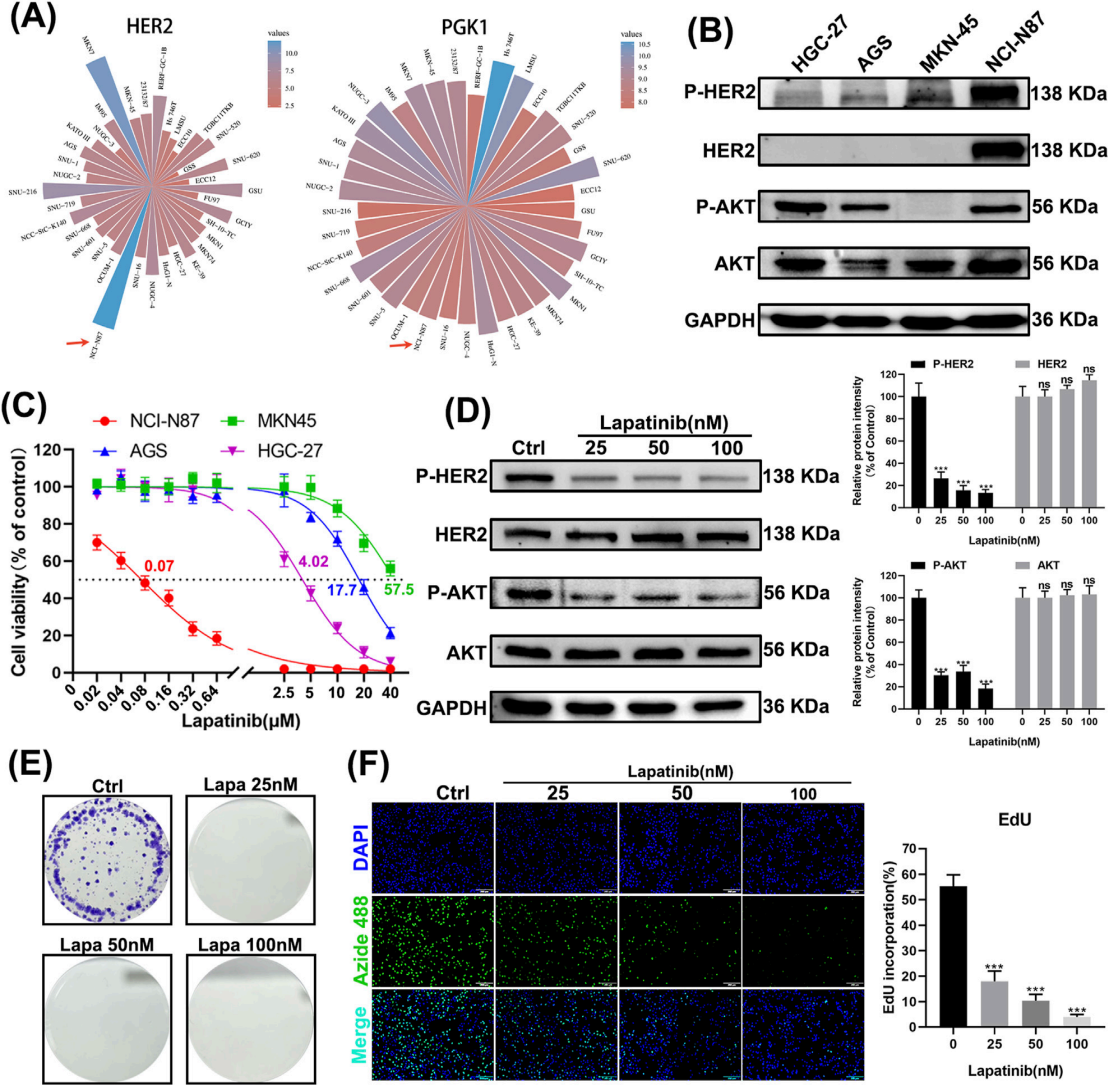
NCI-N87 is HER2-dependent and sensitive to lapatinib. **(A)** Expression levels of HER2 and PGK1 in gastric cancer cell lines according to the CCLE database. **(B)** Western blot images of gastric cancer cell lines HGC-27, AGS, MKN-45, and N87. **(C)** CCK-8 assay results of gastric cancer cell lines HGC-27, AGS, MKN-45, and N87 after 24 h of exposure to lapatinib. **(D)** Western blot analysis of N87 cells after 24 h of exposure to lapatinib. **(E)** Images of clone formation in N87 cells. **(F)** Images of EdU assay in N87 cells. Scale bar, 100 μm.

To validate these findings, we selected a gastric cancer cell line that is HER2-dependent and sensitive to lapatinib. By analyzing the CCLE (Cancer Cell Line Encyclopedia) database, we identified N87 and MKN7 as cell lines with high expression of HER2 and PGK1 (Figure 5A). Western blot experiments confirmed the elevated expression of HER2 protein and its phosphorylated form (p-HER2) in N87 cells, along with the activation of the downstream signaling molecule AKT (p-AKT) (Figure 5B). CCK-8 assays demonstrated that N87 cells are highly sensitive to lapatinib, with an IC_50_ value of 0.07 μM (Figure 5C). Upon exposure of N87 cells to lapatinib, phosphorylated HER2 was significantly downregulated, and the phosphorylation of its downstream effector AKT was inhibited (Figure 5D). Lapatinib also suppressed clone formation in N87 cells (Figure 5E) and inhibited cell proliferation (Figure 5F). These results indicate that N87 is a HER2-addicted cell line and sensitive to lapatinib.

### 3.6 Inhibiting lactate production enhances the sensitivity of N87 to lapatinib

**FIGURE 6:**
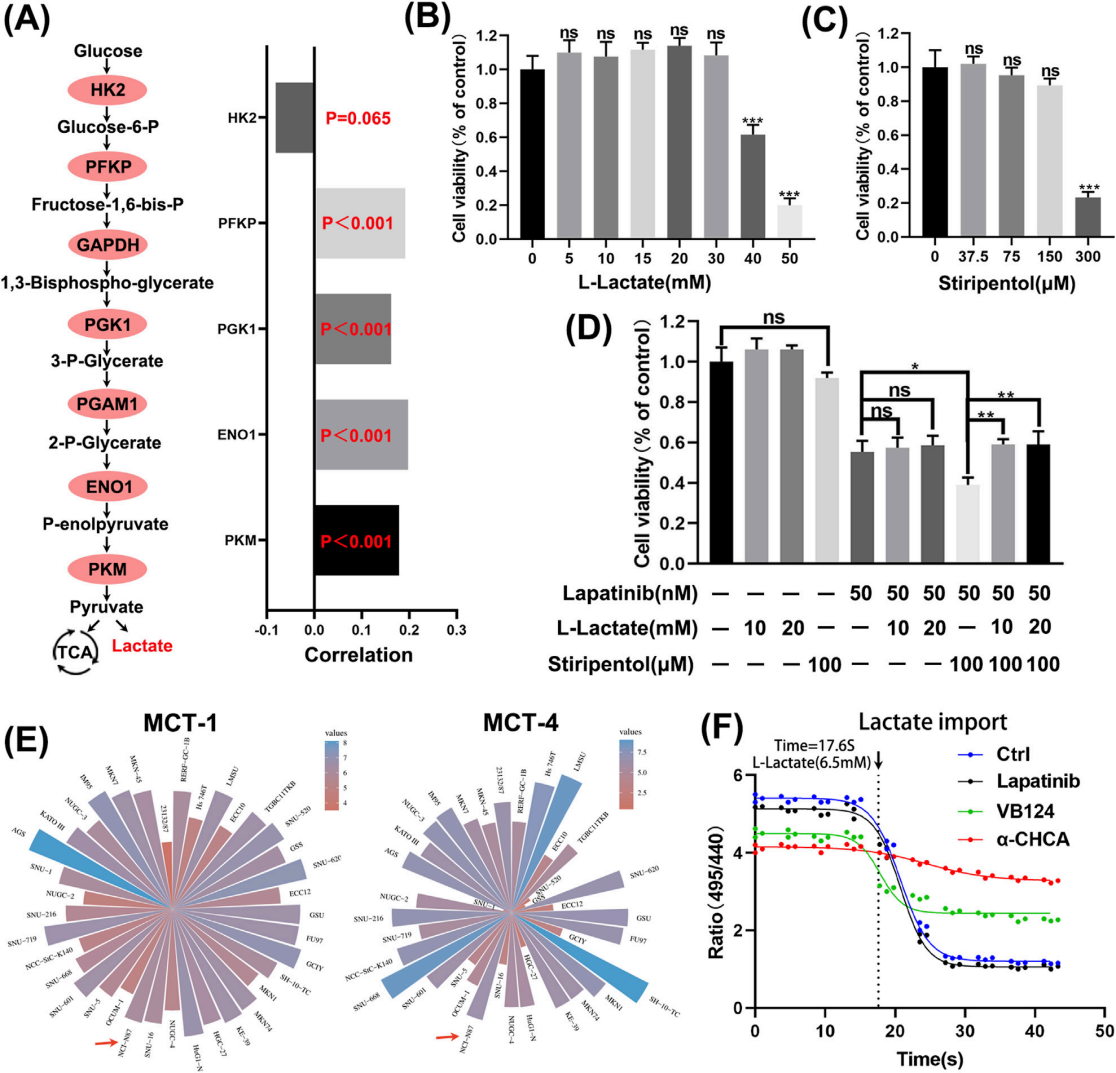
Enhancing N87 sensitivity to lapatinib by inhibiting lactate production. **(A)** Correlation analysis between expression levels of key rate-limiting enzymes in glycolysis and lapatinib sensitivity. **(B,C)** CCK-8 assays of N87 cells exposed to L-Lactate and Stiripentol for 48 h **(D)** CCK-8 experiments with combined applications of lapatinib, L-Lactate, and Stiripentol for 48 h **(E)** mRNA expression levels of MCT-1 and MCT-4 in gastric cancer from the CCLE database. **(F)** Lactate influx experiment with positive controls VB124 (20 μM), an MCT4 inhibitor, and α-cyano-4-hydroxycinnamic acid (α-CHCA) (5 mM), an inhibitor of MCTs.

The aforementioned analysis indicates that PGK1, a crucial rate-limiting enzyme in glycolysis, mediates tumor resistance to lapatinib. We found that most rate-limiting enzymes in glycolysis may mediate tumor cell resistance to lapatinib (Figure 6A). Stiripentol is a lactate dehydrogenase inhibitor (Chen et al., 2024). We selected the optimal concentrations of L-lactate and stiripentol (Figures 6B,C) and discovered that exogenous lactate addition did not rescue the viability of N87 cells exposed to lapatinib, whereas the combination of stiripentol and lapatinib more effectively suppressed cell viability (Figure 6D). Notably, exogenous lactate addition to N87 cells treated with a combination of stiripentol and lapatinib restored cell viability to levels comparable to those observed with lapatinib monotherapy. N87 cells express the monocarboxylate transporters MCT1 and MCT4 (Figure 6E), and lactate influx experiments demonstrated that lactate can readily enter N87 cells (Figure 6F), with VB124 and α-cyano-4-hydroxycinnamic acid (α-CHCA) serving as positive controls. Therefore, the drug-resistant effect of lactate as an exogenous additive is likely exerted through its entry into the cells rather than by binding to membrane receptors on the cell surface. Collectively, these results indicate that low concentrations of lactate can increase the resistance of N87 cells to lapatinib, whereas exogenous addition of higher lactate concentrations does not mediate resistance to lapatinib in N87 cells.

### 3.7 PGK1 mediates resistance to lapatinib in N87 cells

**FIGURE 7:**
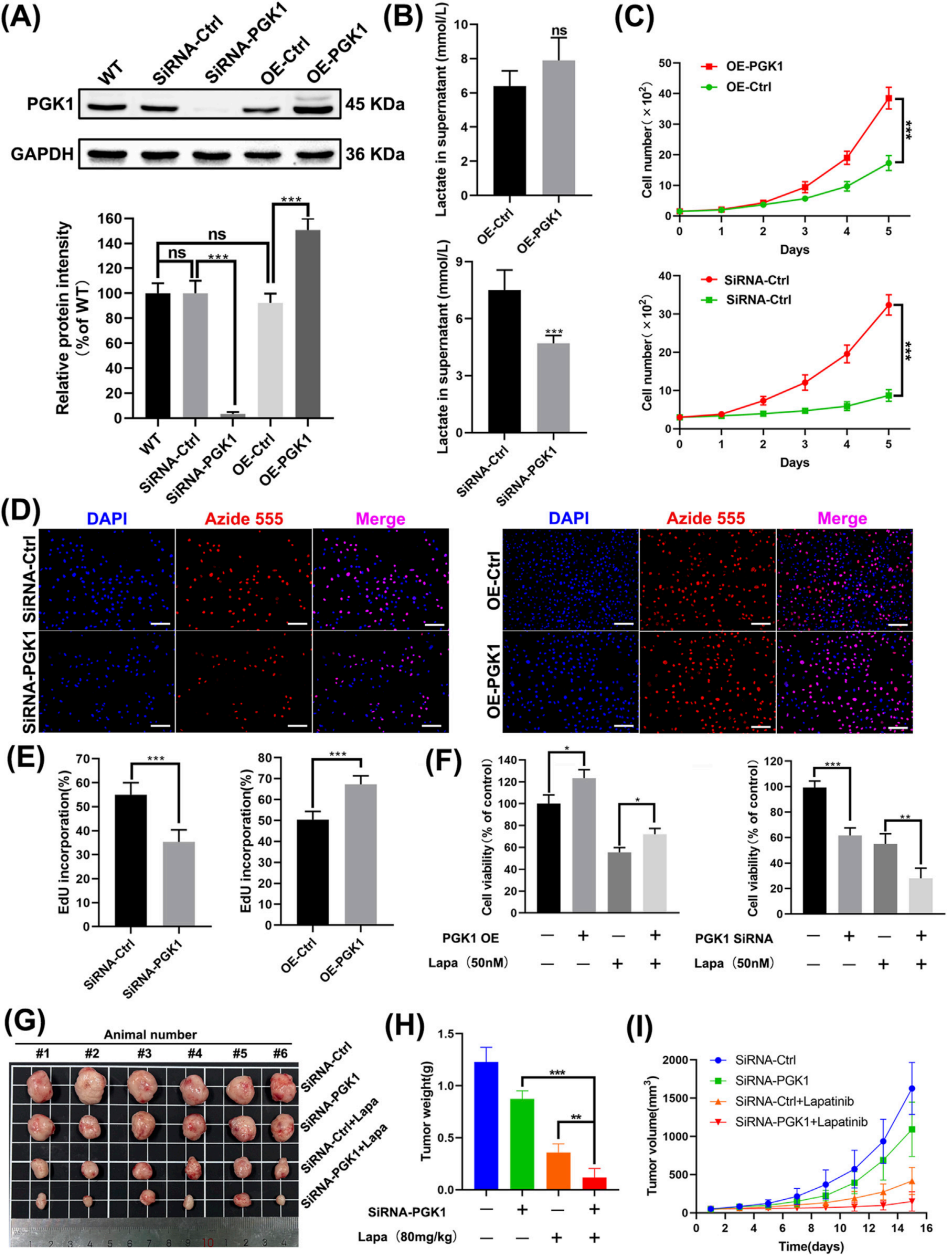
PGK1 mediates resistance to lapatinib in N87 cells. **(A)** Western blot images of N87 cells after knockdown and overexpression treatments. **(B)** Measurement of lactate content in the culture medium. **(C)** Continuous monitoring of cell growth over 5 days using the CCK-8 assay. **(D,E)** Cell proliferation rate assessed by the EdU incorporation. Scale bar, 100 μm. **(F)** N87 cell viability determined by the CCK-8 assay. **(G)** Images of the tumors after isolation. The dosage of Lapatinib was 80 mg/kg. **(H)** Tumor weights on the day of experiment termination. **(I)** Tumor volumes were recorded every 2 days using a vernier caliper.

We constructed cell lines with PGK1 overexpression and knockdown (Figure 7A). Knockdown of PGK1 expression significantly inhibited lactate production, whereas exogenous expression of PGK1 failed to increase lactate production (Figure 7B). The proliferation rate of cell lines overexpressing PGK1 was significantly increased, while the proliferation rate of cell lines with knocked-down PGK1 expression was significantly decreased (Figures 7C–E). Overexpression of PGK1 in N87 cells exposed to lapatinib significantly reversed the proliferation inhibitory effect, whereas knockdown of PGK1 significantly enhanced the inhibitory effect of lapatinib on N87 cells (Figure 7F). Subsequently, we established xenograft tumor models using the aforementioned cell lines. N87 cells with PGK1 knockdown exhibited a significantly slower growth rate *in vivo* compared to the control group. Significantly, when the tumor model with PGK1 knockdown was treated with lapatinib, it exhibited a more pronounced tumor regression compared to the PGK1-knockdown-only group, suggesting that the expression level of PGK1 influences the *in vivo* tumor response to lapatinib (Figures 7G–I). These results indicate that PGK1 mediates resistance to lapatinib in N87 cells.

### 3.8 PGK1 mediates lapatinib resistance in N87 cells via a bypass pathway

**FIGURE 8:**
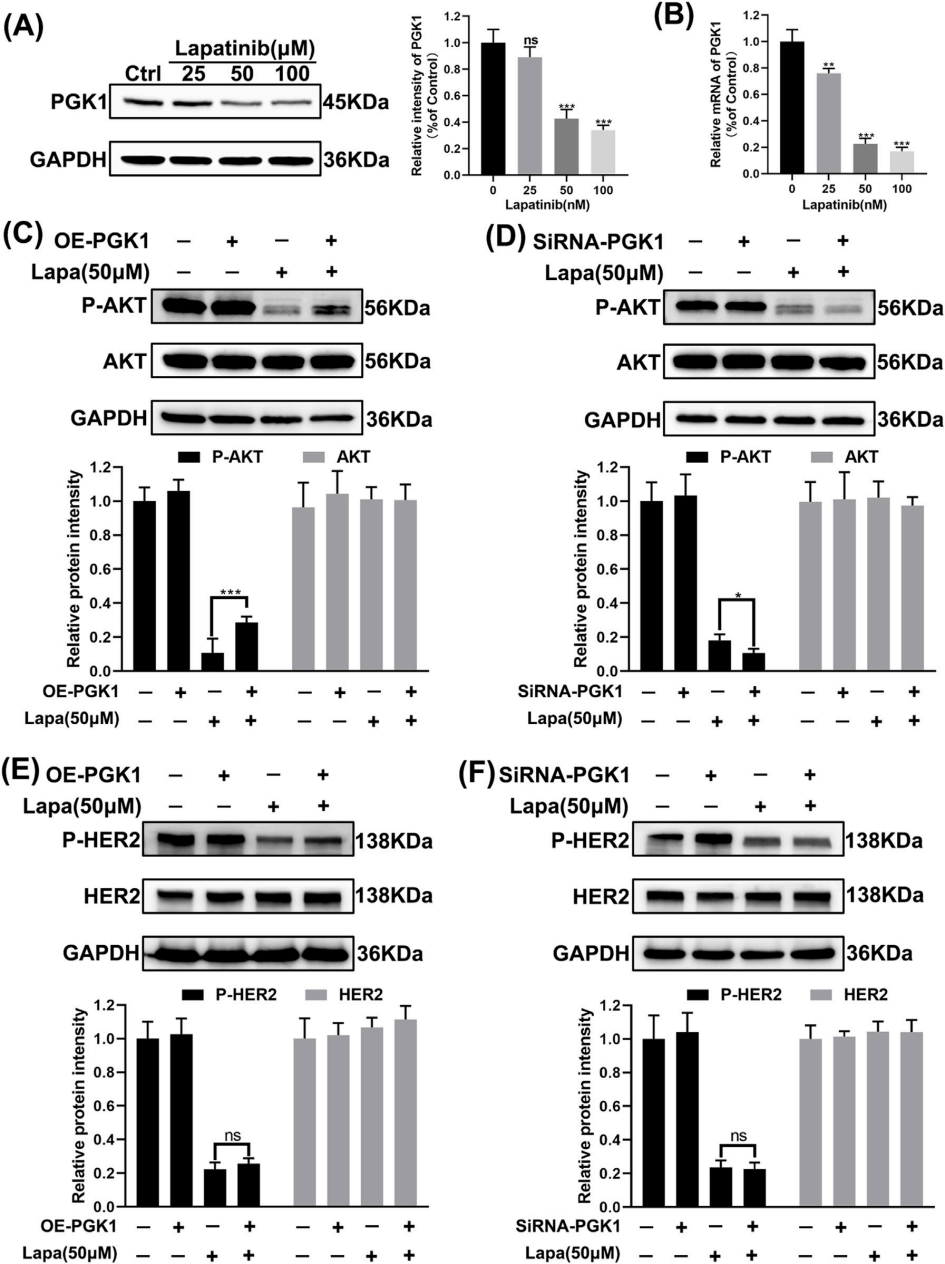
PGK1 mediates lapatinib resistance in N87 cells via a bypass pathway. **(A)** Immunoblot images of N87 cells after 72 h of exposure to lapatinib. **(B)** mRNA levels in N87 cells after 72 h of exposure to lapatinib. **(C–F)** Western blot images of N87 cells subjected to overexpression and knockdown treatments, following 72 h of exposure to lapatinib (50 nM).

We found that the expression of PGK1 protein was significantly downregulated in N87 cells after exposure to lapatinib (Figure 8A), which is demonstrated to be regulated through a transcriptional mechanism (Figure 8B). Overexpression of PGK1 reverses the lapatinib-mediated inhibition of AKT, while knockdown of PGK1 enhances the inhibitory effect of lapatinib on AKT (Figures 8C,D). However, neither overexpression nor knockdown of PGK1 affects the protein level or phosphorylation status of HER2, nor does it influence the efficacy of lapatinib in inhibiting P-HER2 (Figures 8E,F). These results suggest that PGK1 mediates lapatinib resistance in N87 cells via a bypass pathway, leading to the reactivation of downstream AKT signaling.

## 4 Discussion

Gastric cancer remains the second leading cause of cancer-related deaths globally, particularly in East Asia, where it has the highest incidence worldwide (Lordick et al., 2022; Jemal et al., 2010). Approximately 20% of gastric cancer patients exhibit HER2 protein amplification, which is directly associated with aggressive tumor behavior. Although there is currently no consensus on the relationship between HER2 amplification and patient prognosis in gastric cancer (Janjigian et al., 2012; Gómez-Martin et al., 2012; Begnami et al., 2011; Kim et al., 2011), our analysis of multiple GEO datasets suggests that HER2 amplification is associated with shortened patient survival (Figure 4F). Receptor tyrosine kinases like HER2 primarily control tumor survival and proliferation by regulating phosphatidylinositol-3-kinase (PI3K) signaling. Our results support the notion that HER2-addicted N87 cells, upon lapatinib treatment, exhibit significantly reduced phosphorylation levels of AKT, a downstream effector of PI3K signaling, and show suppressed proliferation (Figure 5). Therefore, the application of lapatinib in gastric cancer with HER2 amplification should be considered an effective therapeutic strategy. Clinical studies have shown that trastuzumab combined with chemotherapy for advanced gastric cancer can increase overall survival by 2.7 months compared to chemotherapy alone, making trastuzumab the first molecularly targeted drug approved for gastric cancer treatment (Bang et al., 2010). However, not all patients with HER2 amplification respond to trastuzumab, prompting the development of more HER2-targeted drugs. Unfortunately, lapatinib targeting HER2 in combination with chemotherapy has not demonstrated superior efficacy compared to chemotherapy alone (Satoh et al., 2014). Investigating the mechanisms of lapatinib resistance in HER2-amplified gastric cancer patients may not only provide new therapeutic strategies for HER2-positive patients but also potentially improve their sensitivity to lapatinib and trastuzumab. Identifying predictive biomarkers for lapatinib resistance is therefore essential to guide therapeutic strategies.

Phosphoglycerate kinase 1 (PGK1) is a key rate-limiting enzyme in glycolysis, catalyzing the conversion of 1,3-bisphosphoglycerate to 3-phosphoglycerate and generating one ATP molecule. It not only contributes to lactate production but also serves as one of the primary sources of energy generation in glycolysis (Guo et al., 2024). While studies suggest that PKM2 activity is suppressed in tumors, PGK1 may play a more critical role among glycolysis rate-limiting enzymes in tumorigenesis (Ledford, 2014). Research has indicated that PGK1 enhances glycolysis via CXCR4/ERK pathway activation, which is associated with sorafenib resistance in clear cell renal cell carcinoma (He et al., 2022). Restricting lactate metabolism in aggressive HER2^+^ brain metastatic cells can promote their death or increase the latency of metastasis (Parida et al., 2022). Our data demonstrate that limiting lactate production in HER2^+^ gastric cancer cells increases their sensitivity to lapatinib, while exogenous lactate addition can rescue N87 cells (Figure 6). Simultaneously, the application of high lactate concentrations alone does not contribute to lapatinib resistance in N87 cells. It was indeed unexpected at first that lactate alone could not rescue the inhibitory effect of Lapatinib on N87 cells. However, after the application of the lactate inhibitor stiripentol, which restricted lactate production, the subsequent addition of exogenous lactate was able to rescue the inhibitory action of Lapatinib on N87 cells. We believe that a low concentration of lactate can promote N87 cell resistance to Lapatinib, whereas once the lactate content reaches a certain threshold, further increases in concentration no longer contribute to the development of resistance. Even though overexpressing PGK1 did not increase lactate secretion in N87 cells, PGK1 overexpression still mediated lapatinib resistance in these cells. Therefore, we believe that PGK1 mediates lapatinib resistance in N87 cells through two factors: on the one hand, PGK1 acts as a glycolysis enzyme involved in lactate production, mediating the survival of tumor cells exposed to lapatinib. On the other hand, high PGK1 expression mediates lapatinib resistance in a lactate-independent manner. These events seem unrelated to lapatinib’s target HER2, as modulating PGK1 did not significantly affect HER2 phosphorylation levels. This may be related to the activation of other RTK families, Toll-like receptors (TLRs), etc. Recent studies have shown that HER3 signaling is activated in HER2-positive gastric cancer (GC) patients (Leto et al., 2015). The heterodimer formed by HER3 and HER2 is a key activator of PI3K/AKT signaling in HER2-dependent tumors (Wang et al., 2016). Studies have indicated that in gastric cancer with AKT inhibition, a negative feedback loop can drive the restoration of HER3 signaling, thereby mediating trastuzumab resistance (Gijsen et al., 2010; Narayan et al., 2009). This may also be the reason why lapatinib cannot completely inhibit AKT in cells with high PGK1 expression, ultimately leading to bypass resistance (Figure 8). In an important study a few months ago, it was first revealed that lactylation of the DNA repair protein NBS1 plays a critical role in tumor chemotherapy resistance, and it was proposed that targeting the inhibition of NBS1 lactylation or regulating the lactate metabolism pathway could reverse chemotherapy resistance. Similarly, this study reveals the mechanism by which the key glycolysis-limiting enzyme PGK1 promotes lapatinib resistance in HER2-positive gastric cancer. Regulating PGK1 protein levels or the glycolysis pathway may become a new strategy to improve the sensitivity of HER2-positive gastric cancer patients to lapatinib.

## 5 Conclusion

This study indicates that regulating the expression level of PGK1 impacts the sensitivity of HER2-positive gastric cancer to lapatinib, potentially serving as a therapeutic strategy for HER2-positive gastric cancer patients who do not respond to lapatinib.

## Data Availability

The original contributions presented in the study are included in the article/Supplementary Material, further inquiries can be directed to the corresponding authors.
