# Supplementary material for: Targeting PGK1 as a Novel strategy to regulate the sensitivity of HER2 positive gastric cancer to lapatinib
Source: Front Pharmacol. 2025 Jul 25;16:1530492. doi: 10.3389/fphar.2025.1530492 (PMC12331609; doi:10.3389/fphar.2025.1530492)

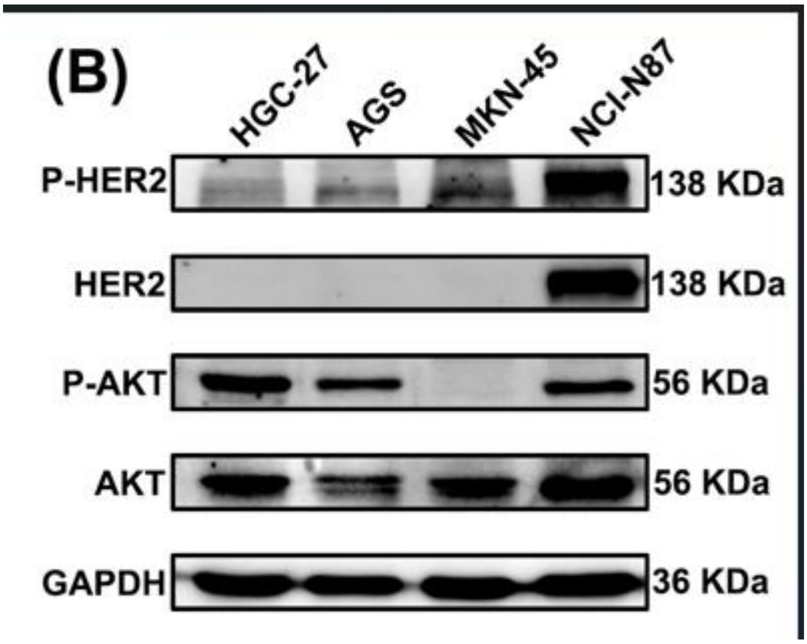

From top to bottom, they are P-HER2, HER-2, P-AKT, AKT, and GAPDH, respectively. The strips on the left and right are duplicates, with the middle and rightmost ones being the Marker.

To improve efficiency, we cut the original membrane. Some bands, such as AKT and GAPDH, have close molecular weights, so they were not separated. We have circled these marks in red to indicate that they originated from the same membrane.

P-HER2

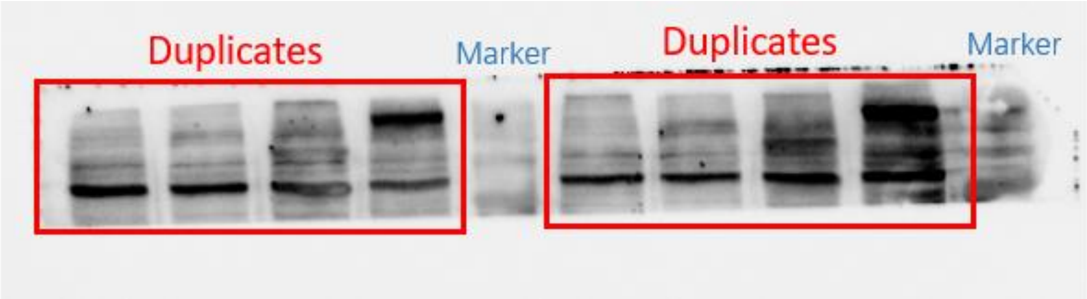

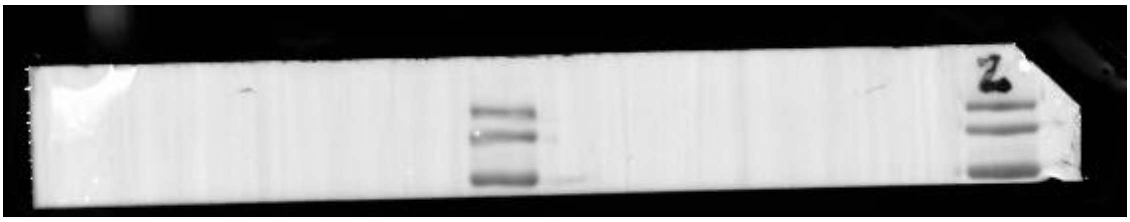

HER-2

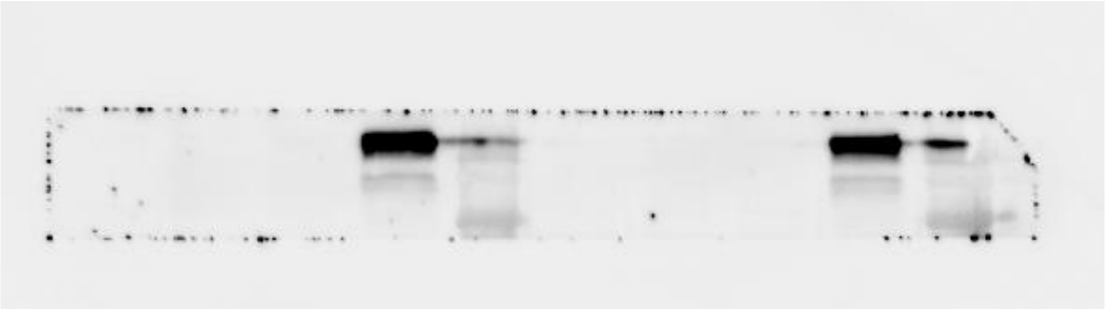

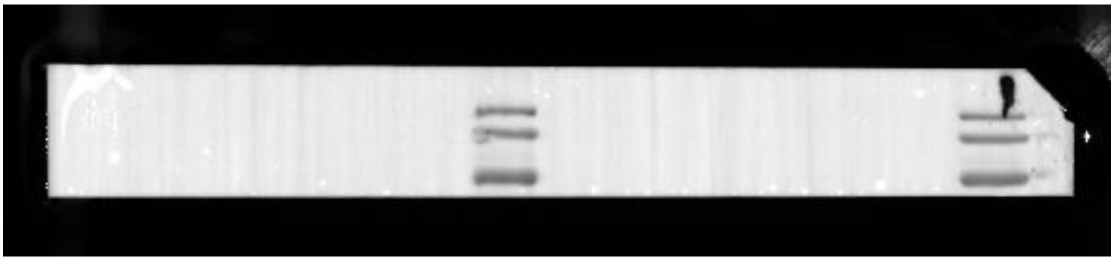

P-AKT

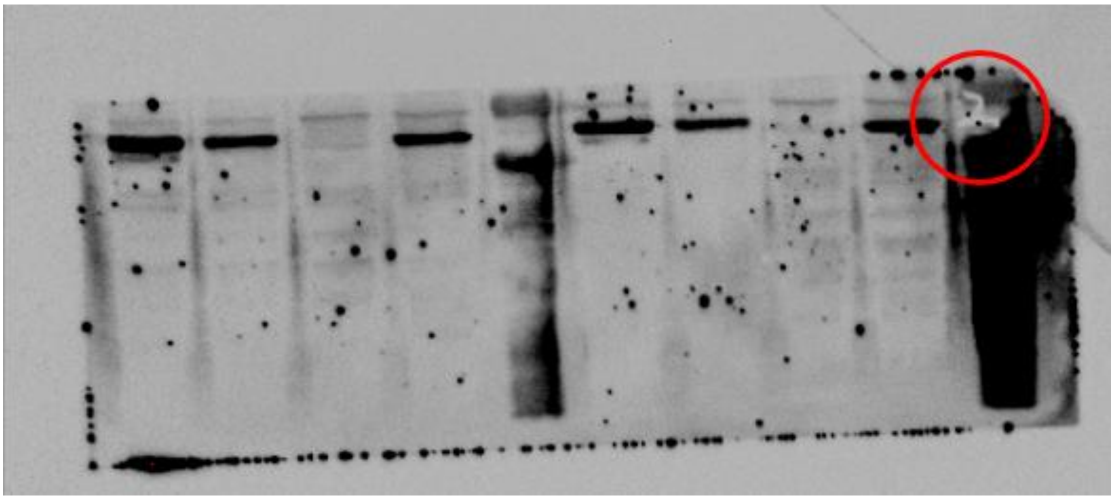

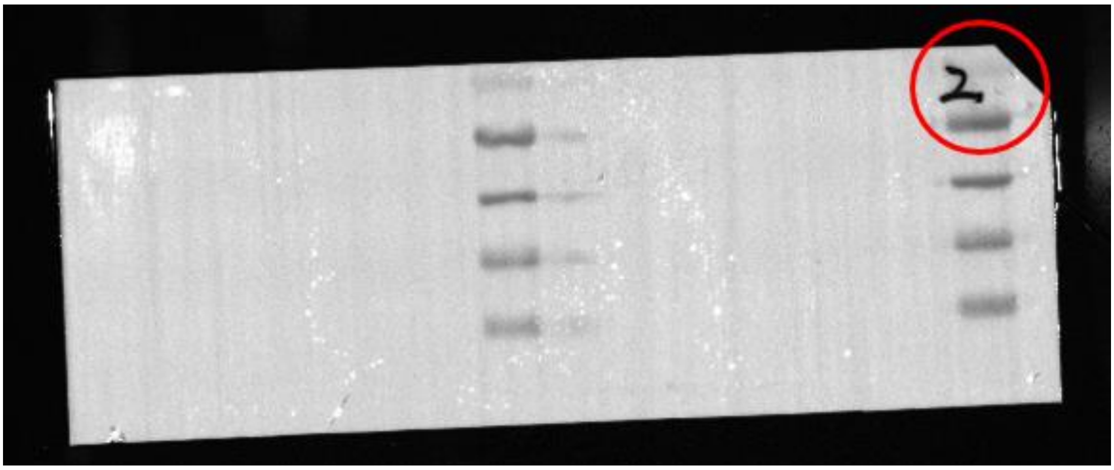

AKT

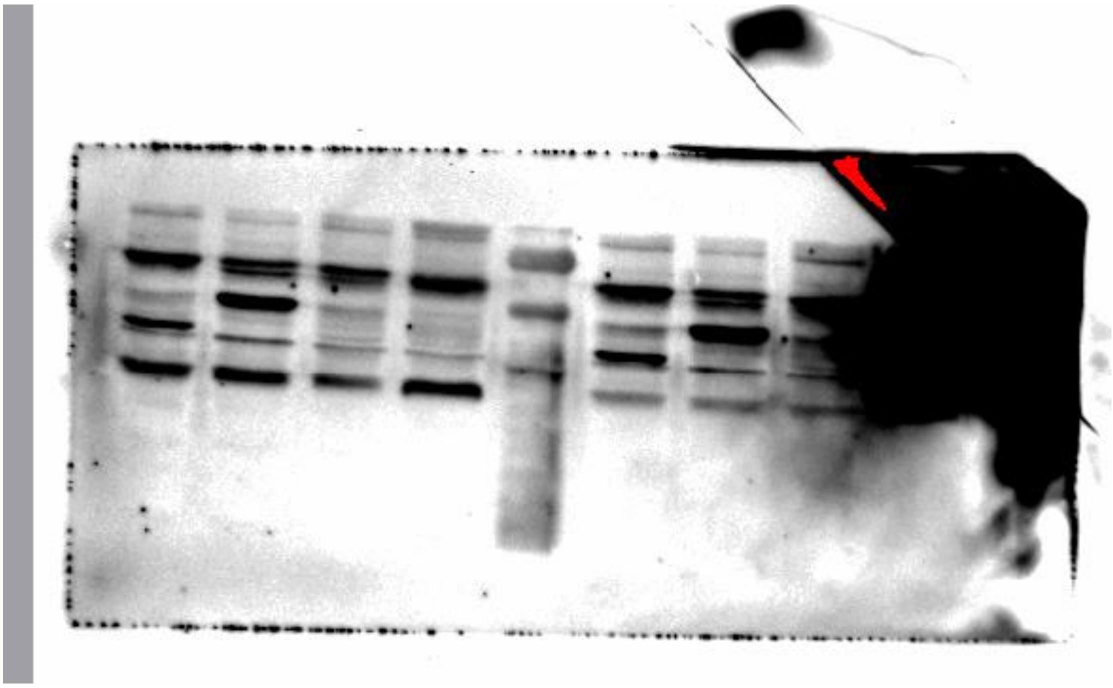

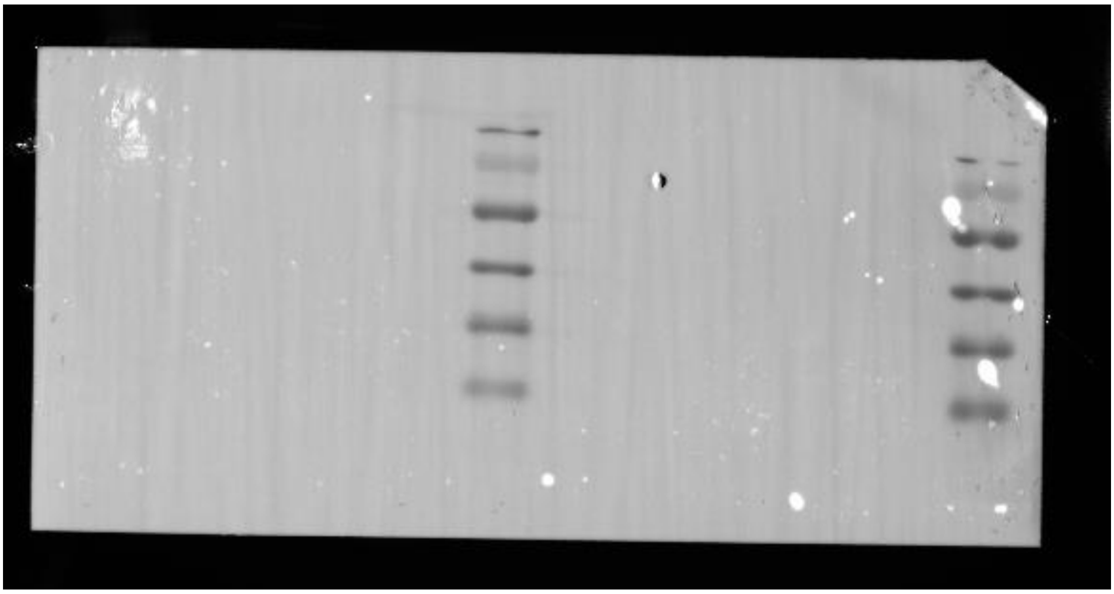

GAPDH

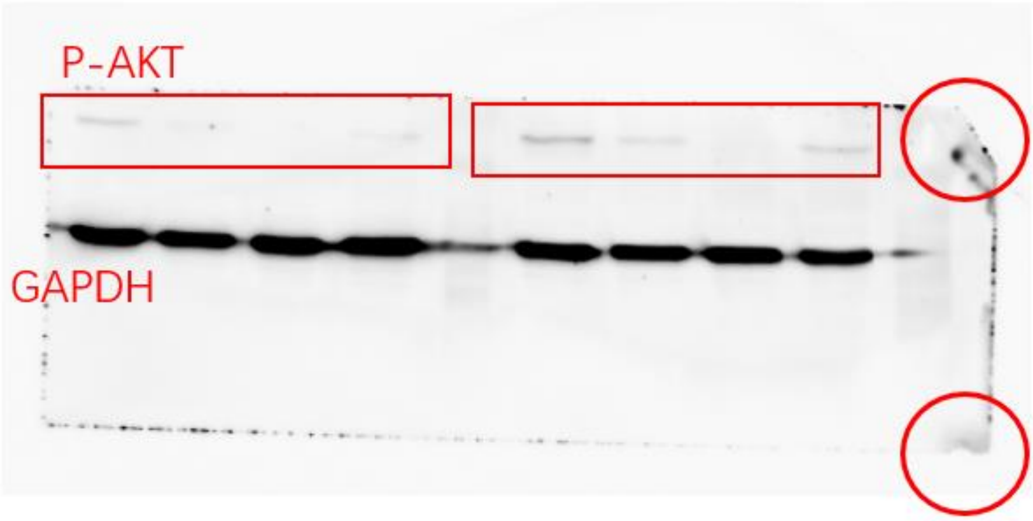

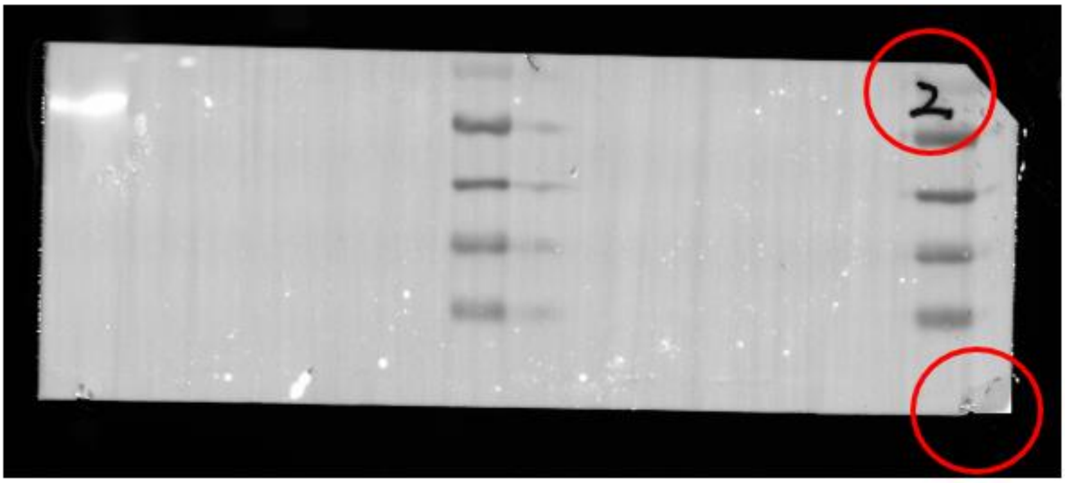

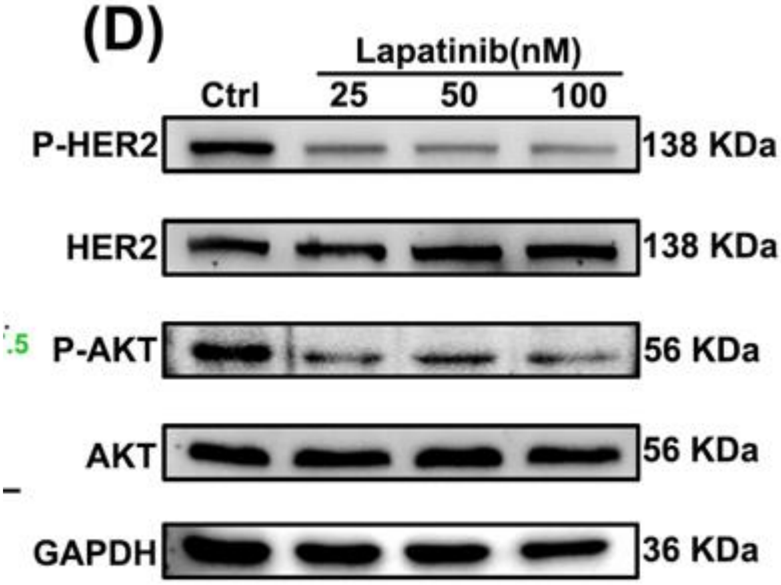
(D)
Lapatinib(nM)
Ctrl
25
50
100
P-HER2
138 KDa
HER2
138 KDa
P-AKT
56 KDa
AKT
56 KDa
GAPDH
36 KDa

P-HER2

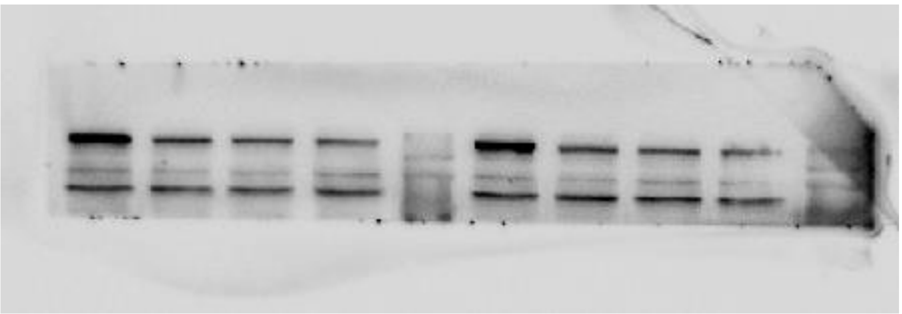

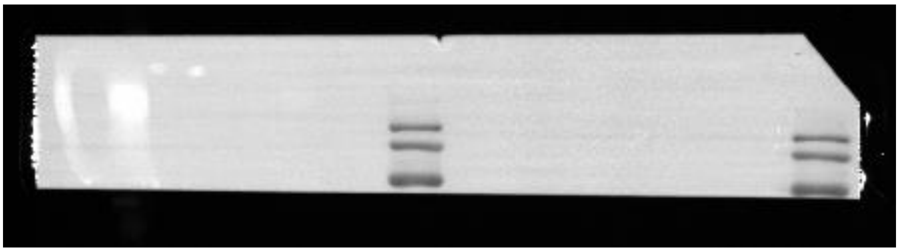

HER-2

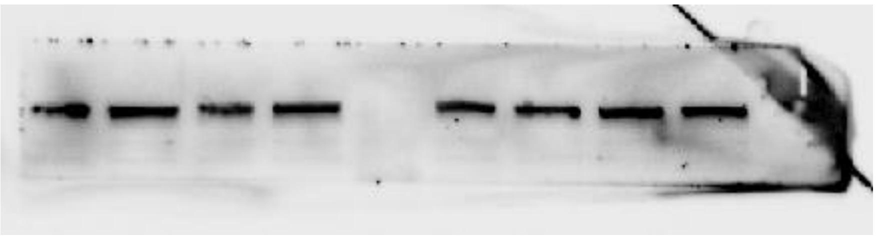

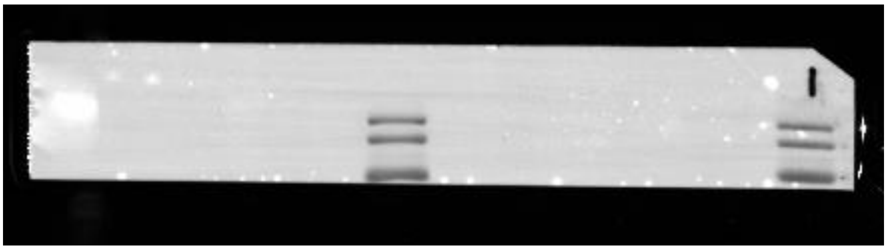

P-AKT

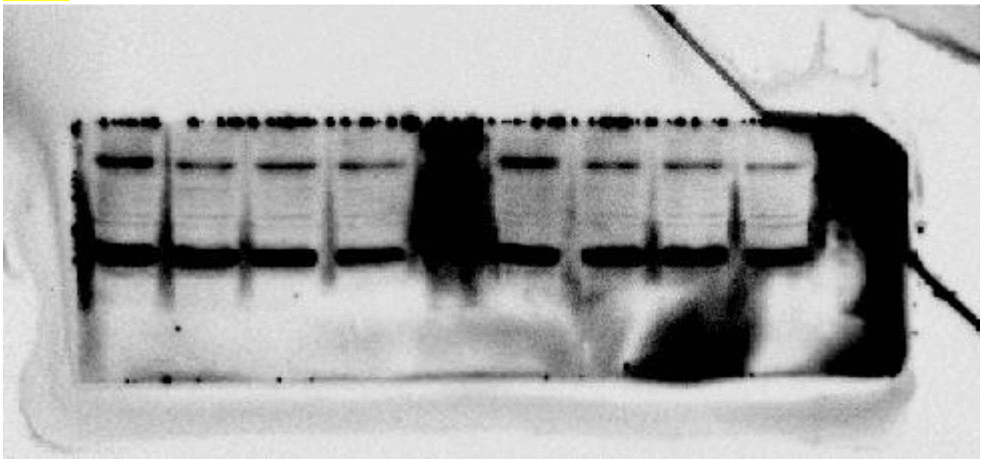

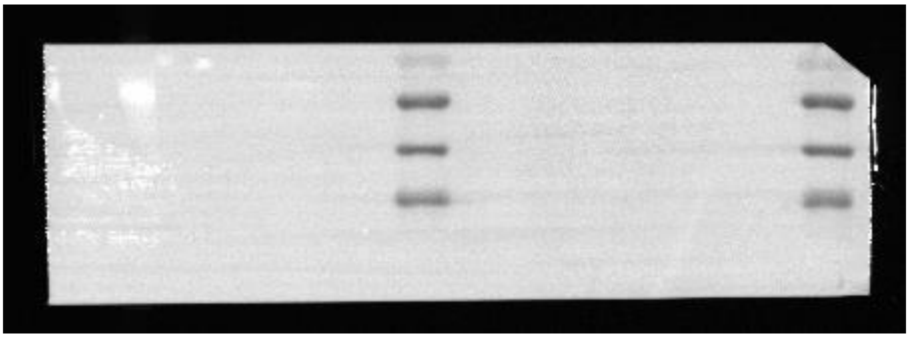

AKT

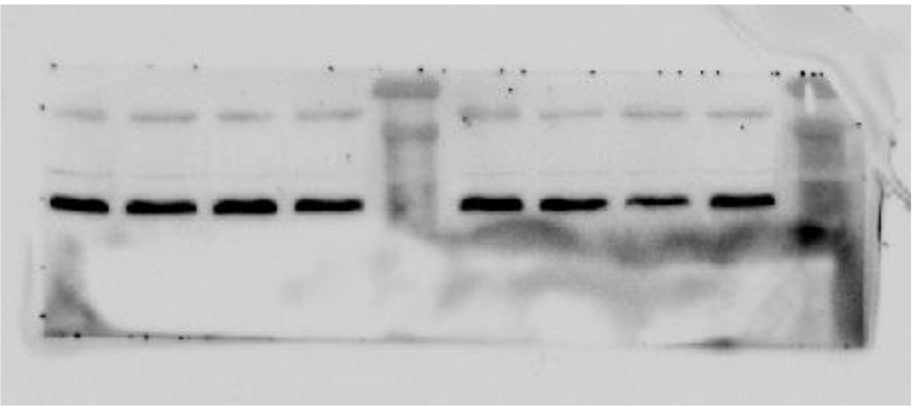

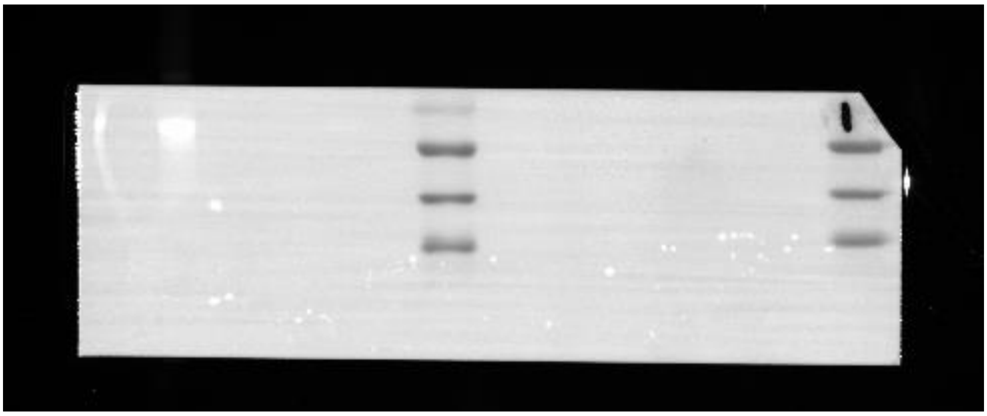

GAPDH

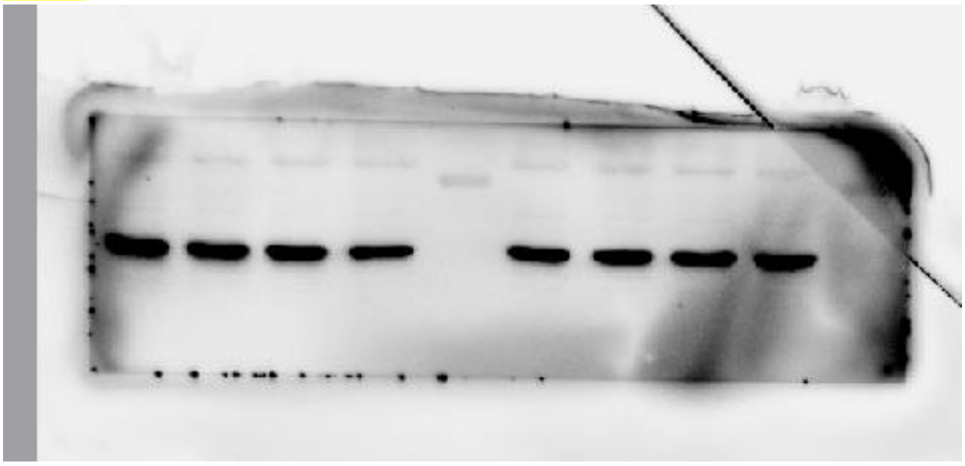

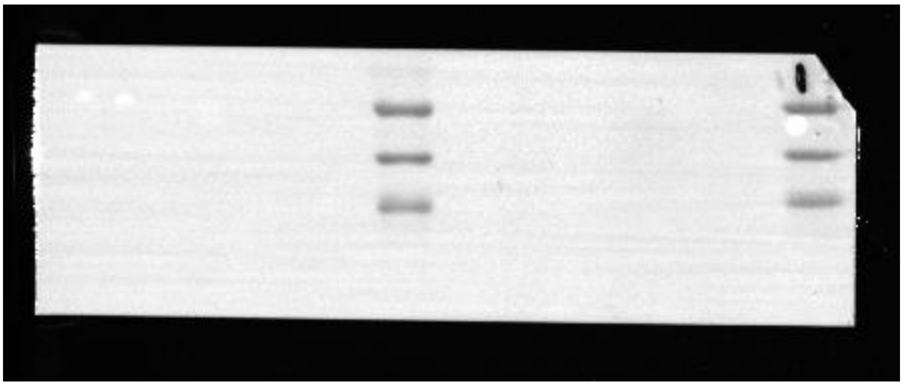

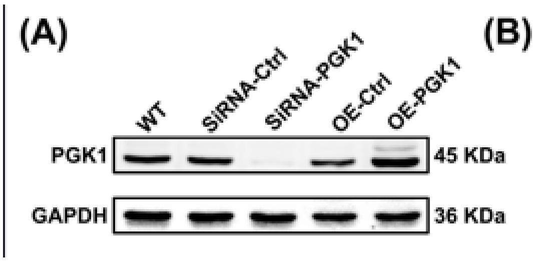
(A)
(B)
WT
SiRNA-Ctrl
SiRNA-PGK1
OE-Ctrl
OE-PGK1
PGK1
45 KDa
GAPDH
36 KDa

PGK1

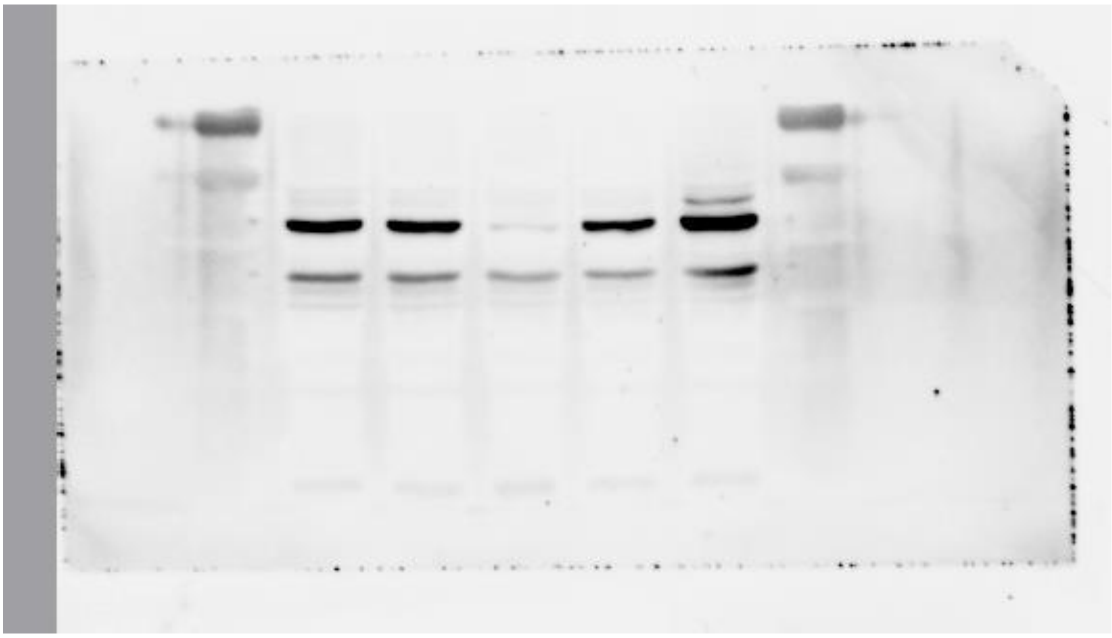

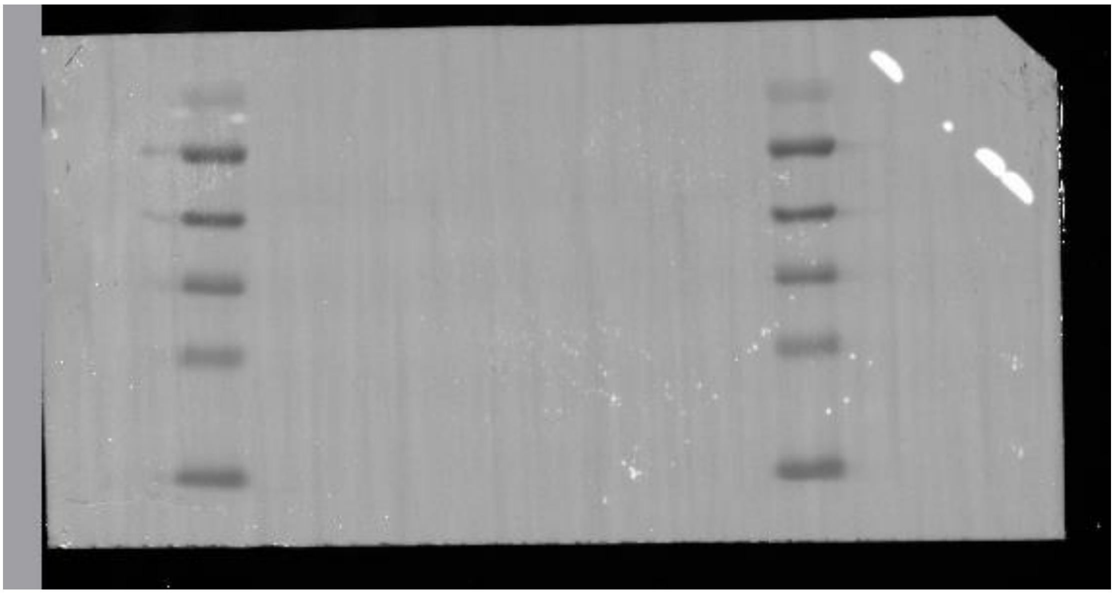

GAPDH

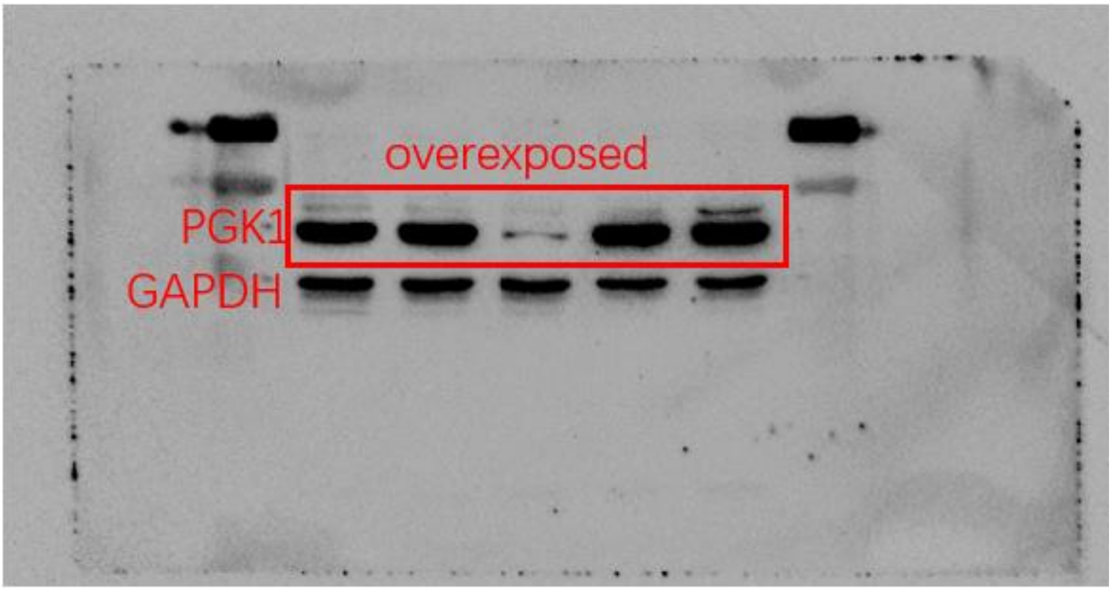

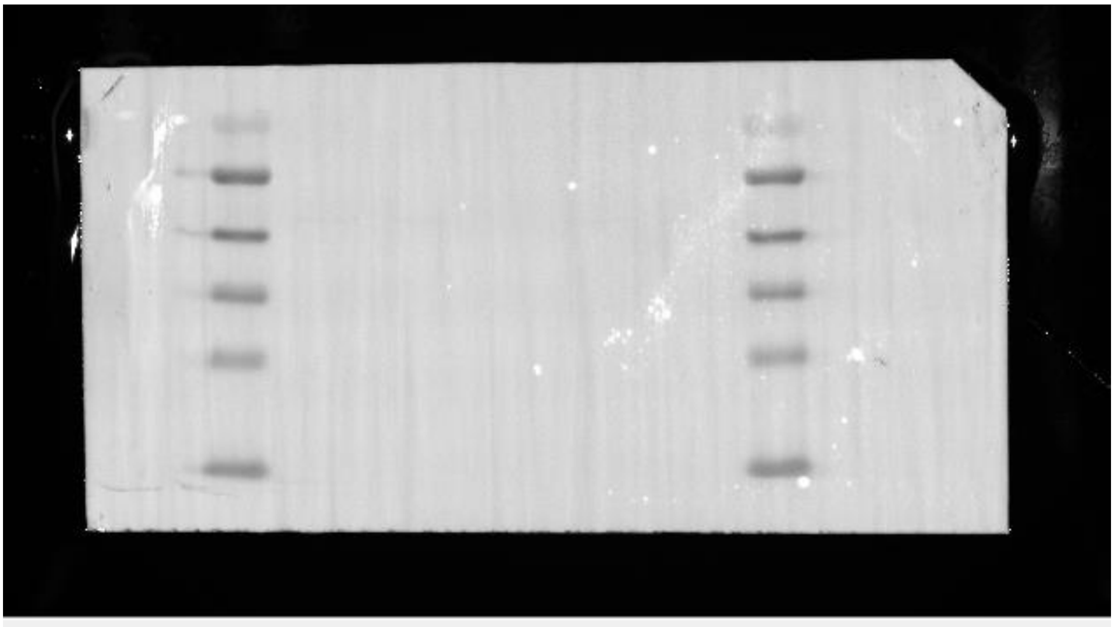

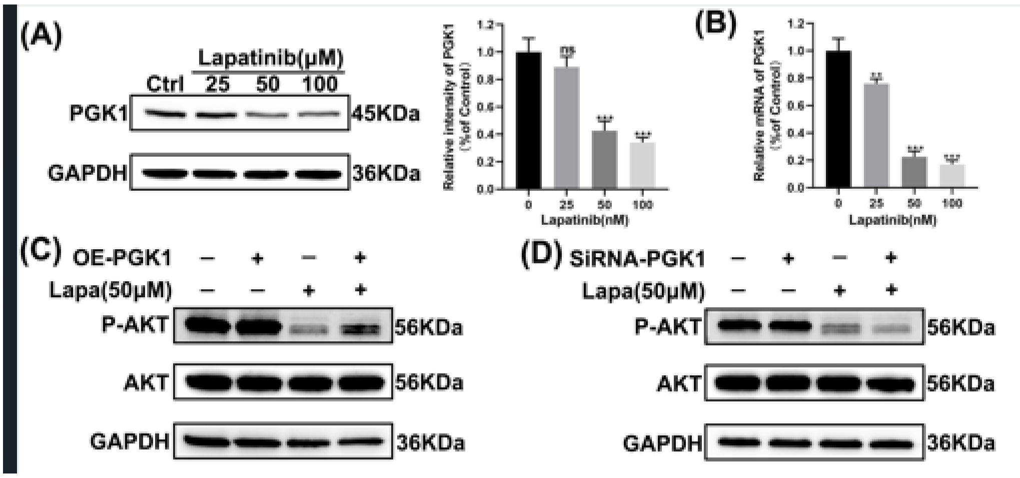
(A)
Lapatinib(μM)
Ctrl 25 50 100
PGK1
45KDa
GAPDH
36KDa
Relative intensity of PGK1 (%of Control)
1.2
1.0
0.8
0.6
0.4
0.2
0.0
ns
***
0 25 50 100
Lapatinib(nM)
(B)
Relative mRNA of PGK1 (%of Control)
**
***
0 25 50 100
Lapatinib(nM)
(C) OE-PGK1 – + – +
Lapa(50μM) – – + +
P-AKT
56KDa
AKT
56KDa
GAPDH
36KDa
(D) SiRNA-PGK1 – + – +
Lapa(50μM) – – + +
P-AKT
56KDa
AKT
56KDa
GAPDH
36KDa

PGK1

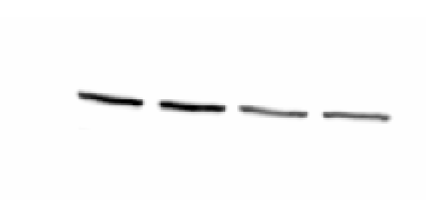

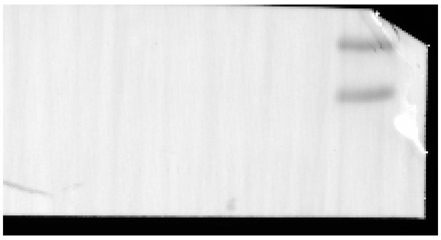

P-AKT

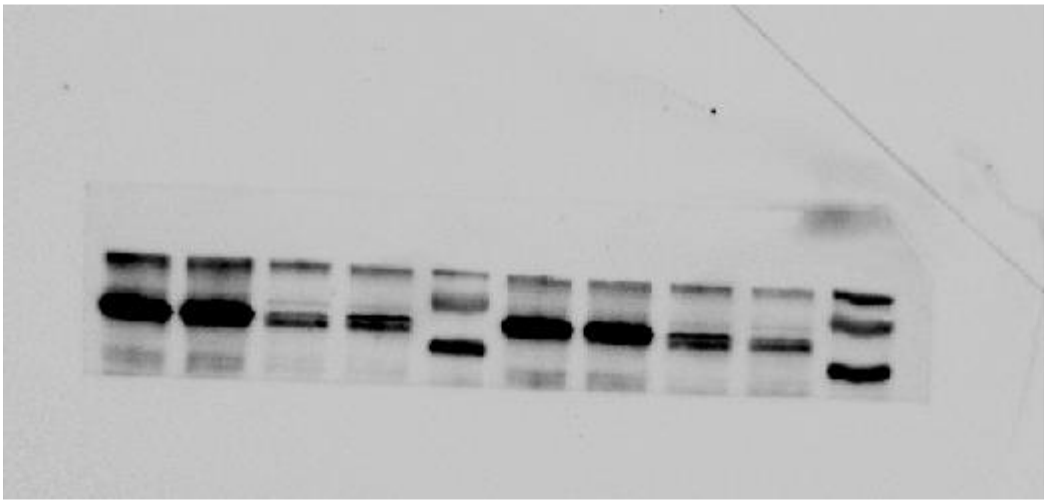

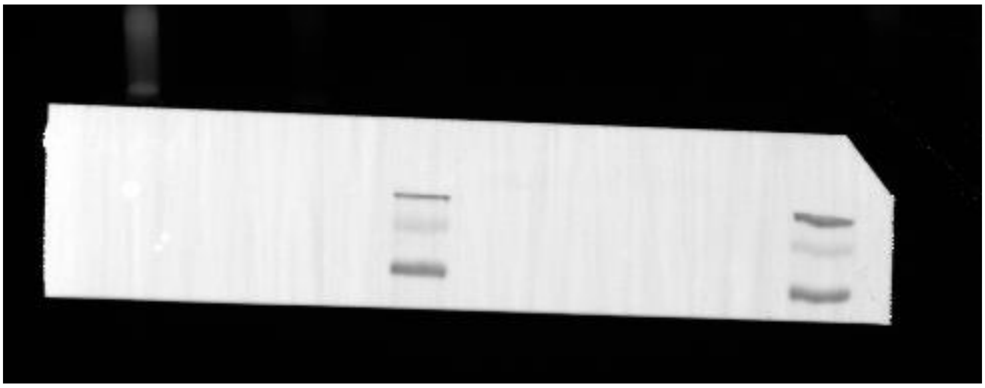

AKT

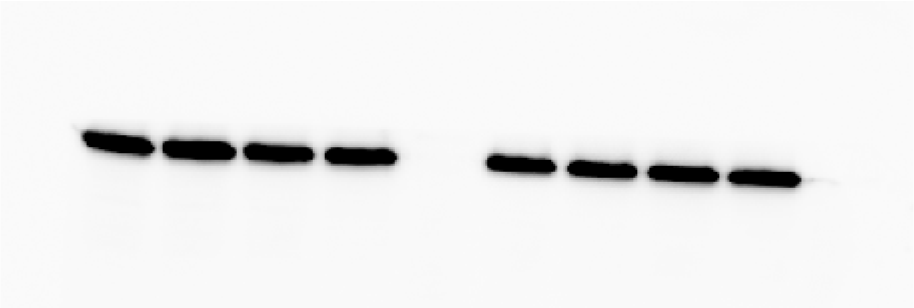

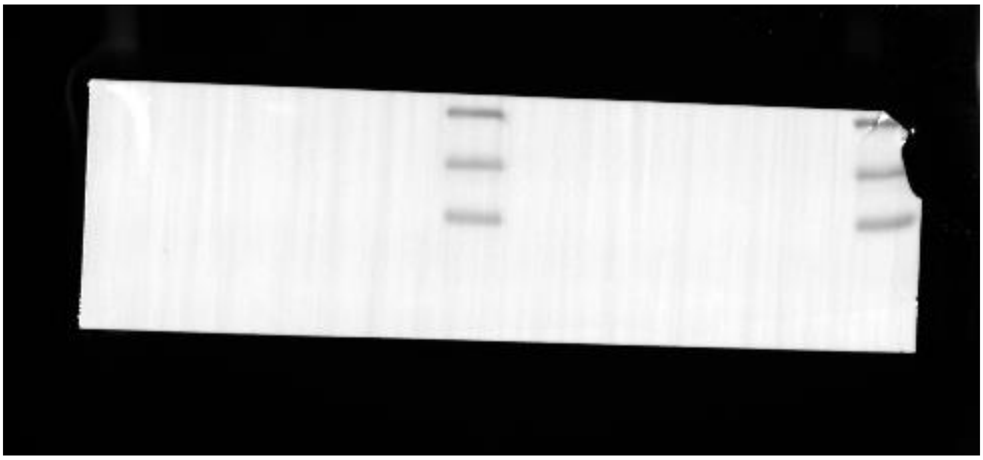

GAPDH

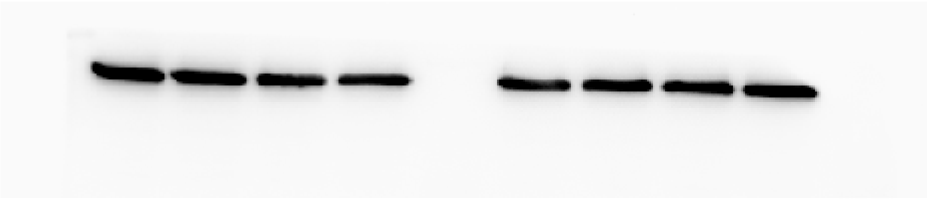

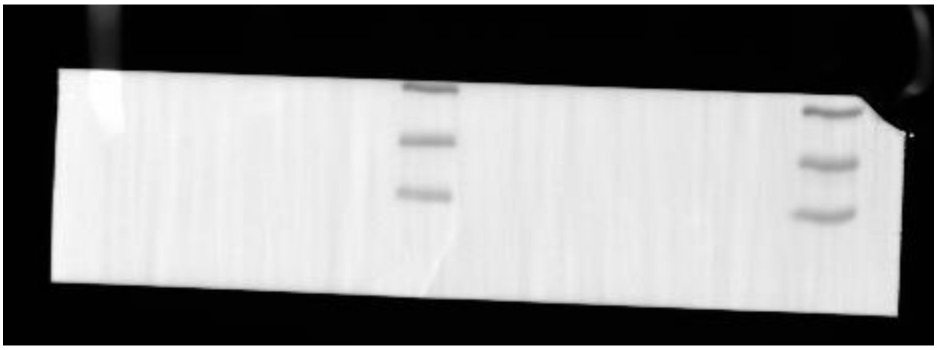

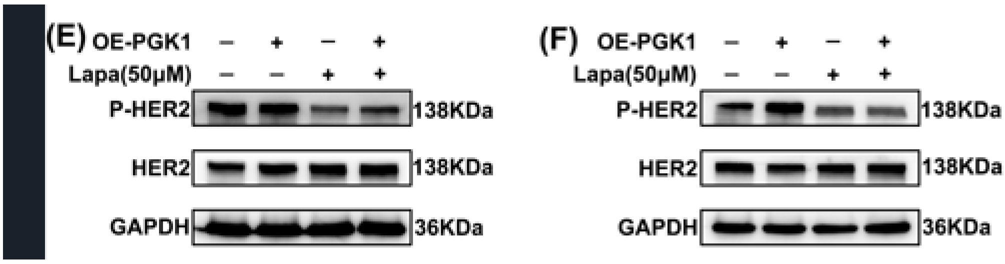
(E) OE-PGK1 – + – +
Lapa(50μM) – – + +
P-HER2 138KDa
HER2 138KDa
GAPDH 36KDa
(F) OE-PGK1 – + – +
Lapa(50μM) – – + +
P-HER2 138KDa
HER2 138KDa
GAPDH 36KDa

P-HER2

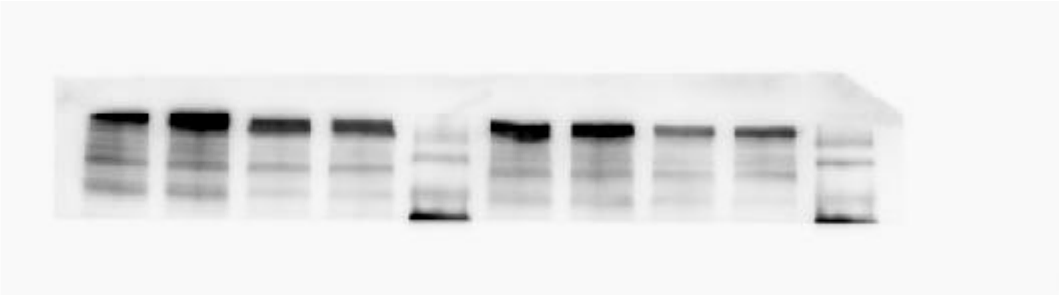

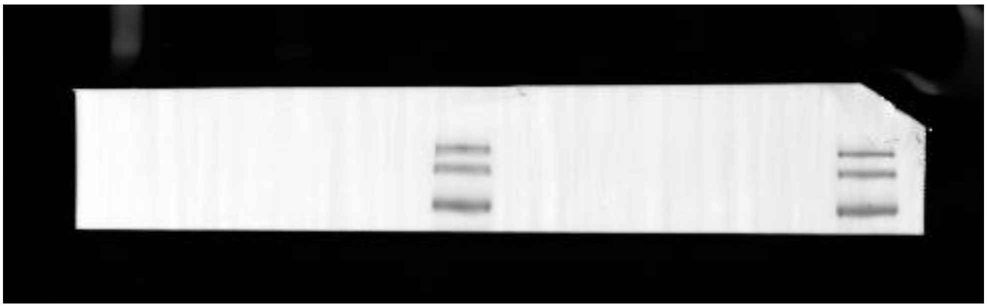

HER-2

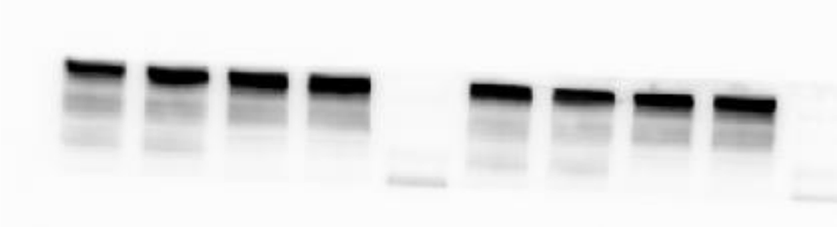

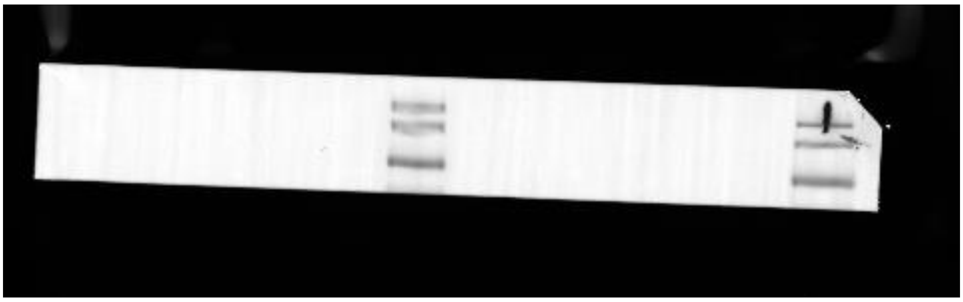

GAPDH

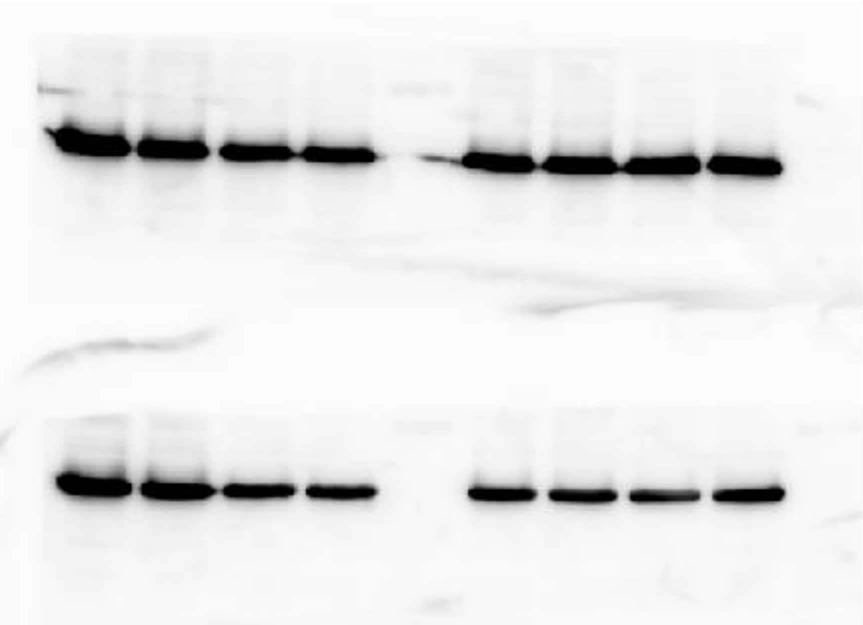

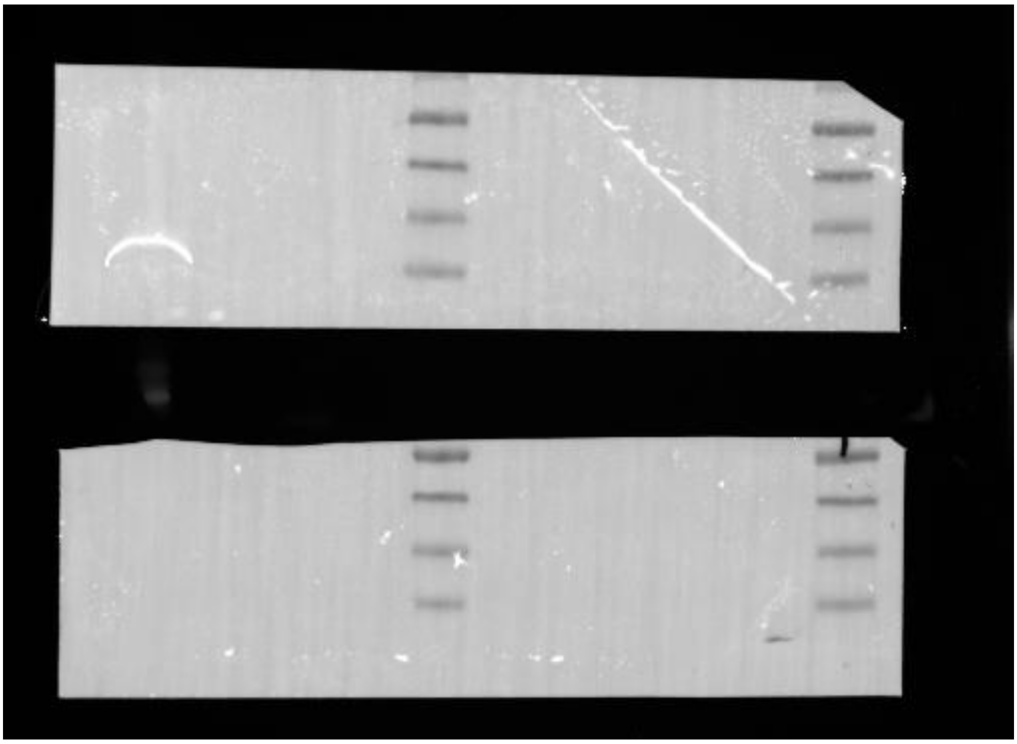

Supplement: Supplementary file 1 [file DataSheet1.pdf]
